# Evaluation of Influencing Factors on Metabolism of Land-Based *n*-3 Poly Unsaturated Fatty Acids—The KoALA Study

**DOI:** 10.3390/nu15204461

**Published:** 2023-10-20

**Authors:** Timo Drobner, Theresa S. Braun, Michael Kiehntopf, Peter Schlattmann, Stefan Lorkowski, Christine Dawczynski

**Affiliations:** 1 Junior Research Group Nutritional Concepts, Institute of Nutritional Sciences, Friedrich Schiller University Jena, 07743 Jena, Germany; timo.drobner@uni-jena.de (T.D.); theresa.braun@uni-jena.de (T.S.B.); 2 Competence Cluster for Nutrition and Cardiovascular Health (nutriCARD) Halle-Jena-Leipzig, 07743 Jena, Germany; peter.schlattmann@med.uni-jena.de (P.S.); stefan.lorkowski@uni-jena.de (S.L.); 3 Institute of Clinical Chemistry and Laboratory Diagnostics, University Hospital Jena, 07747 Jena, Germany; michael.kiehntopf@med.uni-jena.de; 4 Department of Medical Statistics, Informatics and Data Science, University Hospital Jena, 07743 Jena, Germany; 5 Institute of Nutritional Sciences, Friedrich Schiller University Jena, 07743 Jena, Germany

**Keywords:** α-linolenic acid, linseed oil, eicosapentaenoic acid, docosahexaenoic acid, *n*-3 PUFA metabolism

## Abstract

This study aimed to investigate the impact of influencing factors (sex, eicosapentaenoic acid (EPA) status at baseline, linoleic acid (LA) intake, milk fat intake) on the conversion of α-linolenic acid (ALA) obtained from linseed oil into its long-chain metabolites. In addition, the effect of ALA on cardiovascular risk markers was investigated. This study used a parallel design approach by randomly assigning the 134 subjects to one of four diets (high in LA (HLA); low in LA (LLA); high in milk fat (MF); control (Western diet)) each enriched with linseed oil (10 en%, 22–27 mL ≙ 13–16 g ALA). Blood samples were taken at baseline and after 4, 8, and 12 weeks of dietary intervention. The study was fully completed by 105 subjects (57.4 ± 12.1 years; 65.7% female). Results showed that ALA (296–465%), C-20:4n3 (54–140%), and EPA (37–73%) concentrations in erythrocytes increased in all groups (*p* < 0.01). In contrast, docosahexaenoic acid (19–35%, *p* < 0.01) and *n*-3 index (10–21%, *p* < 0.05) dropped in the HLA, LLA, and control groups. An increase in C-22:5n3 was only observed in the MF (36%) and control groups (11%) (*p* < 0.05). In addition, an increase in LA (7–27%) was found in the HLA, LLA, and control groups, whereas C-20:3n6 (16–22%), arachidonic acid (10–16%), C-22:4n6 (12–30%), and C-22:5n6 (32–47%) decreased (*p* < 0.01). The conversion into EPA was higher in men than in women (69 vs. 39%, *p* = 0.043) and in subjects with low EPA status compared to participants with high EPA status (79 vs. 29%, *p* < 0.001). A high LA status attenuates the conversion rate. In line with the literature, no clear effects on blood lipids and parameters of glucose metabolism were found in relation to ALA supplementation.

## 1. Introduction

Cardiovascular diseases (CVDs) are among the greatest threats to human health worldwide [1]. In Germany, CVDs are the primary cause of death, accounting for 33% of all cases [2]. Age, sex, and genetic disposition are non-controllable risk factors. However, avoiding modifiable factors such as smoking, physical inactivity, obesity, and an unhealthy diet have been shown to support prevention of and therapies for CVDs. The importance of dietary patterns to the occurrence of CVDs is indisputable. In particular, an increased intake of whole grains, nuts, seeds, fruits, and *n*-3 polyunsaturated fatty acids (PUFA)-rich seafoods as well as a reduced sodium intake can reduce the risk of cardiometabolic deaths [3].

The traditional Western diet in Germany is characterized by a high intake of energy, simple carbohydrates, and refined starch, as well as saturated fats, with an inadequate consumption of monounsaturated fatty acids (MUFA), PUFA, and dietary fiber [4]. This dietary pattern is associated with increased cardiovascular risk [5]. In addition, the ratio between polyunsaturated *n*-6 and *n*-3 fatty acids of about 7–9:1 in the German diet exceeds the current recommendations of the German Nutrition Society (DGE) of an *n*-6 PUFA/*n*-3 PUFA ratio of about 5:1 [6]. The cardioprotective potential, especially in secondary prevention, of the long-chain *n*-3 PUFA (*n*-3 LC-PUFA) eicosapentaenoic acid (EPA) and docosahexaenoic acid (DHA) from marine sources has already been shown in numerous studies [7,8,9]. Discussed mechanisms for these benefits are their effects on membrane structure, signal transduction, gene expression and formation of mediators (eicosanoids and oxylipins), which are important for regulation of inflammation [10,11,12]. International organizations (ISSFAL, EFSA, FAO) recommend a daily intake of 250–500 mg EPA + DHA [13,14,15]. However, the average EPA + DHA intake worldwide via seafood is only 163 mg/d, with considerable regional differences [16]. While the intake in the Nordic countries is 350–550 mg/d, the intake in Central Europe is below the recommended minimum intake of 250 mg/d. In America, Africa, and South Asia, the daily intake is below 150 mg [16]. Another relevant *n*-3 PUFA is α-linolenic acid (ALA), found in plant-based foods, especially in linseeds, chia seeds, and walnuts. However, its cardioprotective effect seems weaker than that of the marine *n*-3 PUFA EPA and DHA [8,9,12,17,18,19,20].

In general, the human organism can synthesize the long-chain metabolites EPA and DHA from ALA using elongases and desaturases. However, the conversion rate to EPA and DHA is limited to 8% and 0.02–4%, respectively [21]. Furthermore, the same enzyme system is used to convert *n*-6 PUFA linoleic acid (LA) into its long-chain metabolites [22]. Consequently, we hypothesized an inhibition of simultaneous high LA intake on the conversion of ALA into *n*-3 LC-PUFA. Further influencing factors such as age and sex are described [22]. In addition, the milk fat-specific short- and medium-chain, as well as the branched-chain and conjugated fatty acids, could influence the conversion of ALA into *n*-3 LC-PUFA [23,24,25,26].

The present study aims to investigate the changes in the fatty acid distribution of erythrocyte lipids by evaluating the influence of simultaneous LA intake, milk fat intake, sex, and *n*-3 LC-PUFA status at baseline on the metabolism of ALA into its long-chain metabolites.

The influence of background diet on the conversion of ALA from linseed oil into the long-chain metabolites EPA and DHA was studied by implementation of defined menu plans (high in LA (HLA); low in LA (LLA); high in milk fat (MF)). The comparison was made against a control group who followed a typical Western diet. In addition to fatty acid distribution, the impact on cardiovascular risk factors was also investigated.

## 2. Materials and Methods

### 2.1. Study Design (Screening)

The KoALA study was conducted as a randomized, single-center intervention study in parallel design (four arms: defined consumption of linseed oil; differing background diets for three groups using prepared menu plans; one control group; the figure in Section 2.3). The study was conducted in Jena in East Germany between March and June 2018.

In early 2018, participants were recruited using flyers and press releases. The flyers were distributed in public facilities, medical practices, and pharmacies in Jena.

The following inclusion criteria were applied:

- Women (in menopause) and men (50% each), aged between 40 and 65 years, body mass index (BMI) < 30 kg/m^2^.
- Moderately elevated low-density lipoprotein (LDL) cholesterol (>3 mmol/L).
- Consumption of a traditional “Western diet” composed of meat, sausage, dairy products, cereals, vegetables, fruits, etc.
- Stable eating habits at least one year before enrollment.
- No antihypertensive medication or stable dose for >3 months prior to the start of the study and during the entire study period.

The following exclusion criteria were applied:

- Acute or chronic diseases which could affect the results of the present study.
- Use of medication, including systemic glucocorticoids or lipid-lowering medication.
- Use of dietary supplements, incl. multivitamins, fish oil capsules, minerals and trace elements (three months before and during the entire study period).
- Weight loss or weight gain (>3 kg) during the last three months before the study began.
- Relevant food allergies (e.g., milk, nuts).
- Pregnancy or lactation.

Adherence of the subjects to the dietary habits as defined (Western diet, stable eating habits before enrollment, renunciation of dietary supplements) was verified by a personal interview and a questionnaire.

**Figure 1:**
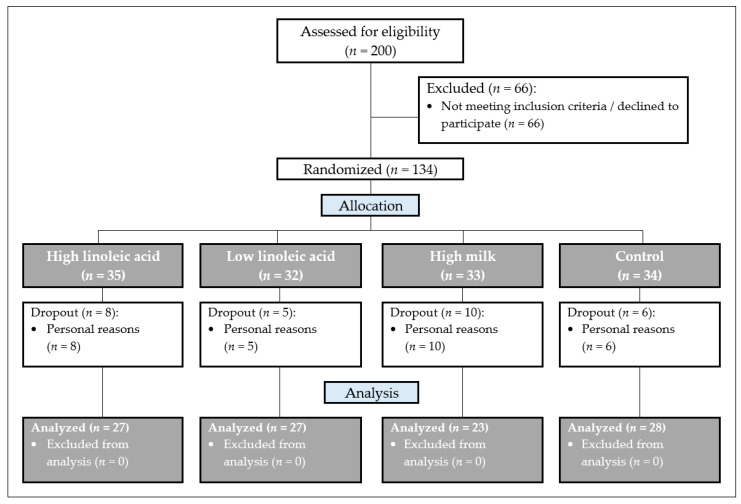
Flowchart diagram of the study population in the different phases of the study. A total of 200 subjects were screened for eligibility. In total, 66 subjects had to be excluded, so that 134 subjects were randomized to the four diet groups. After completion of the study, sorted by group, 27, 27, 23 and 28 participants were included in the statistical analysis.

Before the preliminary phase, 200 subjects were screened for enrollment and 134 participants met the inclusion criteria (Figure 1).

Following the informed consent and after confirmation of the inclusion and exclusion criteria, participants were scheduled for the baseline assessment.

Participants could be withdrawn from the study at any time after enrollment for the following reasons: at the patient’s request, due to severe infections, or if patient compliance with the study protocol was in doubt. The study was conducted in accordance to the Helsinki Declaration of 1975, as revised in 1983. The study protocol was approved by the Ethical Committee of the Friedrich Schiller University Jena (protocol code 5419-01/18) and registered by ClinicalTrails.gov (NCT03558776).

### 2.2. Baseline Assessment

To record and document the habitual dietary patterns within and between groups, the preliminary phase of the KoALA study included full self-reporting of the individual’s dietary intake over seven days. The dietary record was based on the template “Freiburger Ernährungsprotokoll Standard”, which was provided by PRODI software version 6.4 (Nutri-Science, Stuttgart, Germany) and includes foods and usual portion sizes of a typical German diet. Foods not listed in the template were documented manually by the participants, including the name and the amount of the consumed food. The daily energy and nutrient intake was calculated by the software package PRODI.

In addition, participants filled out questionnaires to assess lifestyle, health, and disease status (including medication use).

### 2.3. Study Diet—The KoALA Concept

**Figure 2:**
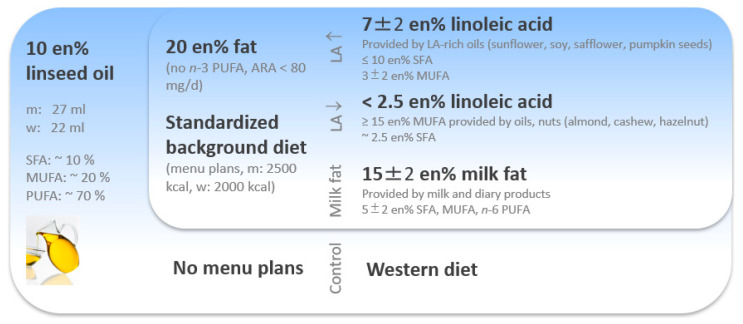
Study design of the KoALA study. Abbreviations: ARA, arachidonic acid; LA, linoleic acid; MUFA, monounsaturated fatty acids; PUFA, polyunsaturated fatty acids; SFA, saturated fatty acids.

After one week of the preliminary phase, each participant was randomly assigned to one of the four groups. The intervention phase of the study ranged over 12 weeks for each participant. Visits to the study center took place at baseline and after 4, 8, and 12 weeks, to take samples, provide study materials and solve potential problems owing to the implementation of the menu plans (Figure 2).

The daily intake of linseed oil constituted 27 mL for men and 22 mL for women. This dosage corresponds to around 10% of the daily energy intake (en%). In addition, the HLA, LLA, and MF groups received defined menu plans for each study day to modify the background diet and homogenize the energy intake. Participants in the control group were asked to maintain a typical Western diet (without menu plans). They also consumed the pre-defined amount of linseed oil.

For the HLA group, the menu plans ensured an additional 20% of daily energy intake via fat which consisted of 7 ± 2 en% LA, up to 10 en% saturated fatty acids (SFA) and 3 ± 2 en% MUFA. The menu plans included oils rich in *n*-6 PUFA such as sunflower, soy, safflower, and pumpkin seeds to ensure these criteria.

For the LLA group, the additional 20 en% of fat consisted of below 2.5 en% LA, at least 15 en% MUFA provided by olive oil and nuts such as almond, cashew, and hazelnut plus around 2.5 en% of SFA.

For the MF group, the additional 20 en% of fat mainly was provided by milk fat from milk and dairy products (15 ± 2 en%) plus 5 ± 2 en% SFA, MUFA, and *n*-6 PUFA.

All menu plans provided 2500 calories for men and 2000 for women, split into 55 en% of carbohydrates, 15 en% of protein, and 30 en% of fat. In addition, the menu plans were characterized by the following criteria:

- Absence of other foods providing *n*-3 PUFA, like fish (oil) and algae (oil).
- Reduced intake of monosaccharides and increased intake of dietary fiber.
- Reduction in salt.
- Increased consumption of fruits and vegetables.
- Reduction in foods which are highly processed, calorie-rich, and low in nutrients, such as fast food and convenience products.

The menu plans included up to five meals per day split into breakfast, lunch, dinner, and up to two snacks. For each meal, detailed information on the type and amount of the food was provided. Recipes for meals that required preparation were also included.

To increase compliance, linseed oil was provided to each participant. The fatty acid distribution for the linseed oil is shown in Table 1.

### 2.4. Sample Collection, Biochemical Analyses, and Further Measurements

Parameters measured as part of the study were biochemical parameters (fasting lipid profile, high-sensitivity c-reactive protein (CRP), apolipoproteins A1 and B, lipoprotein(a), homocysteine, fasting glucose and insulin, glycated hemoglobin A1c (HbA1c)), erythrocyte fatty acid distribution, anthropometric parameters (body weight and composition, blood pressures, heart rate), and micronutrient status (vitamins, minerals, trace elements) (Table S1).

Blood was taken by venipuncture between 7:30 AM and 10:30 AM after at least 12 h overnight fasting. Furthermore, consumption of alcohol and excessive physical activity was not allowed on the day before and on the morning before the venipuncture.

Fasting peripheral venous blood samples were centrifuged (10 min, 2.762 g, 4 °C) to separate plasma and serum. Study parameters were analyzed immediately after blood sampling or by using serum and plasma aliquots stored at –80 °C until the analysis. Samples were prepared according to standard operation procedures. Many of the study parameters were analyzed at the Institute of Clinical Chemistry and Laboratory Diagnostics, Jena University Hospital using an Abbott Architect CI 16200 analyzer (Abbott, Wiesbaden, Germany), HPLC (Shimadzu, Kyoto, Japan) or with regard to HbA1c using the Tosoh HLC-723G11 (Sysmex, Norderstedt, Germany) according to the manufacturer’s recommendations (Table S1). Dianovis performed the analysis of malondialdehyde-modified (MDA)-LDL cholesterol and biotin using an enzyme-linked immunosorbent assay as well as of apolipoprotein A1 and B using COBAS INTEGRA 400 plus System (Roche Diagnostics Ltd., Rotkreuz, Switzerland, Table S1). The Institute of Nutritional Sciences, Friedrich Schiller University Jena, analyzed fatty acid distribution in erythrocyte lipids. Fat was extracted using the Folch and Bligh and Dyer procedure [27,28]. Afterwards, the extracted lipids were saponified and methylated [29]. The success of the methylation was confirmed by separation on silica gel aluminum plates. The resulting fatty acid methyl esters (FAME) were analyzed via gas chromatography (GC-17V3; Shimadzu, Duisburg, Germany). Quantification of each FAME was performed using LabSolutions LCGC software version 5.92 by Shimadzu. The fatty acids were identified using retention times based on previously measured standards for each fatty acid. FAME are always presented in relation to the total FAME content.

The same trained nurse always performed the anthropometric parameters, blood pressure, and heart rate measurements with participants barefoot and in light clothing (single measurement). Body weight was measured in kilograms to the first decimal place and height and waist circumferences to the nearest half centimeter. Waist circumference was measured midway between the lower rib margin and the iliac crest (a thumb’s breadth above the navel). Arterial blood pressure was measured with the subject in a sitting position using the upper arm after the subject had been seated for at least 10 min.

Calibrated instruments were used (scale with integrated stadiometer: seca813; Hamburg, Germany; ergonomic tape measure: seca212; Hamburg, Germany; automatic blood pressure device: boso-medicus uno; BOSCH + SOHN, Jungingen, Germany). Body composition was assessed by a Body Impedance Analyzer (Data Input, Pöcking, Germany; exactness of measurement: 0.5% of measurement value (reactance)/±2.0% of measurement value (resistance)).

### 2.5. Statistical Methods

The power calculation was based on the data from Dittrich et al. (2015) [30]. In a randomized, double-blind, placebo-controlled crossover study, the authors investigated the influence of regular consumption of vegetable oils rich in *n*-3 PUFA on cardiovascular risk factors in patients with moderately elevated triglyceride levels (*n* = 51). The intervention groups received linseed oil (7 g ALA/d), echium oil, or microalgae oil for 10 weeks. The placebo group received sunflower oil (LA group; 10 g LA/d). Consumption of linseed oil increased EPA concentrations in erythrocyte lipids from 0.96 ± 0.35% FAME to 1.26 ± 0.38. The effect of LA content in the background diet on ALA conversion to EPA was calculated using data from Liou et al. (2007) [31] and MacIntosh et al. (2013) [32].

The global power was calculated based on a linear model (analysis of variance) to compare the EPA concentrations at the end of the study for the four groups. The power was 91.1%, assuming a standard deviation of 0.35 and a significance level of 5%.

Contrasts are considered in the linear model mentioned above for power calculation for single comparisons. The most considerable difference in EPA concentration is expected between the HLA and LLA groups or the LLA and MF groups. Based on these data, a group size of 37 subjects has 81% power to achieve an EPA difference of 0.2% FAME between the LLA and control groups (EPA difference between μ1 = 1.2% FAME; μ2 = 1.3% FAME), assuming that the standard deviation is 0.3. In addition, a group size of 37 subjects has 99% power to achieve an EPA difference of 0.3% FAME between the HLA and LLA groups (EPA difference between μ1 = 1.1% FAME; μ2 = 1.4% FAME), assuming that the standard deviation is 0.3. The number of cases was planned using SAS proc power version 9.4 (SAS Institute, Cary, NC, United States). Based on the power calculation with the described data, 37 subjects per group were to be enrolled. Due to the lack of availability of suitable subjects in the recruitment period, only 134 instead of the planned 148 subjects could be included (Figure 1).

For assigning the participants to the four groups, a randomization list was generated with the statistical software R version 3.5.2 (The R Foundation for Statistical Computing, Vienna, Austria).

The primary endpoint of the study was the change in EPA (% FAME) in erythrocyte lipids. Secondary endpoints were the effects on blood lipids, markers of glucose metabolism, micronutrient status, and anthropometric parameters.

For statistical analyses, the statistical software IBM SPSS Statistics version 28.0.1.1 (14) (IBM Germany, Ehningen, Germany) was used. If the data followed a normal distribution, this was tested with the Shapiro–Wilk test. Differences between groups were assessed using one-way ANOVA for normally distributed variables or the Kruskal–Wallis test for not normally distributed variables. Differences within groups comparing baseline and weeks 4, 8, and 12 were assessed using ANOVA for repeated measurements for normally distributed variables or the Friedman test for not normally distributed variables. Fisher’s least significant difference test was performed as a post-hoc test and calculated *p*-values were adjusted manually using the Benjamini–Hochberg procedure [33]. Differences within groups only comparing baseline and week 12 were assessed using paired *t*-test for normally distributed variables or Wilcoxon signed rank test for not normally distributed variables. Correlation analyses were performed using Pearson correlation (normally distributed data) or Spearman’s rank correlation (not normally distributed data).

For calculations of the sum of *n*-3 PUFA, the following *n*-3 PUFA were included: C-18:3 (ALA), C-20:4 (eicosatetraenoic acid, ETA), C-20:5 (EPA), C-22:5 (docosapentaenoic acid, DPA), and C-22:6 (DHA). The sum of *n*-6 PUFA comprises of the *n*-6 PUFA: C-18:2 (LA), C-18:3 (γ-linolenic acid), C-20:2, C-20:3, C-20:4 (arachidonic acid, ARA), C-22:4 and C-22:5. The *n*-3 index was calculated by using the sum of C-20:5 (EPA) and C-22:6 (DHA) in erythrocyte lipids.

We divided our study collective according to EPA status at baseline (<0.9% FAME vs. ≥0.9% FAME) and LA change (% FAME) throughout the study period (<1.5% FAME vs. ≥1.5% FAME) to evaluate the influence of these factors on the conversion of ALA into its long-chain metabolites. The limits represent the cut-off values of each 50% of our participants to compare groups of equal size, as no reference values are available.

## 3. Results

### 3.1. Baseline Characteristics of the Study Collective

The baseline characteristics of the subjects (*n* = 105) who completed the study are shown in Table 2. The collective was composed of approx. 66% women (*n* = 69) and approx. 34% men (*n* = 36). Participants were 57 (±12) years old and had a median BMI of 26 (23, 29) kg/m^2^, corresponding to the defined inclusion criteria. In addition, selected cardiovascular risk factors are shown, with a mean LDL cholesterol of 3.6 (±0.95) mmol/L (according to the defined inclusion criteria).

The distribution for sex and age per group is shown in Table 3. A total of 27 people participated in the HLA, 27 in the LLA, 23 in the MF and 28 in the control group. The distribution of sex ranged from at least 26% to a maximum of 44% for men per group. Accordingly, the distribution for women ranged from 56% to 74%. Groups had a mean age between 56 (±12) years and 59 (±13) years, with no difference between groups.

The 7-day dietary self-report in the week before the start of the intervention showed a comparable intake of energy and macronutrients, selected carbohydrate sources, fatty acids and micronutrients between the four groups (Table S2).

### 3.2. Changes in Fatty Acid Distribution in Erythrocyte Lipids within the Diet Groups

The 12-week intervention resulted in a median increase in ALA by 296–465%, in ETA by 54–140%, and in EPA by 37–73% in each group (*p* < 0.01; Table S3).

An increase in DPA concentration was only observed in the MF (36 ± 44%) and control groups (11 ± 15%) (*p* < 0.05). The HLA (3.2% FAME (2.51, 3.44)) and LLA groups (2.97% FAME (2.29, 3.44)) started with significantly higher DPA concentrations compared to the MF (2.43% FAME (2.01, 2.81)) and control groups (2.36% FAME (2.22, 2.72)).

In contrast, the DHA concentration, which represents the main *n*-3 fatty acid in erythrocytes, fell by a median of 19–35% in the HLA, LLA, and control groups (*p* < 0.01). The reduction was greater in the HLA and LLA groups compared to the MF and control groups (*p* < 0.05; Table S3). However, at baseline, the HLA (5.73% FAME (4.66, 6.54)) and LLA groups (5.2% FAME (4.4, 5.98)) had significantly higher starting values than the MF group (4.18% FAME (3.06, 4.95)) and the HLA group in addition to the control group (4.76% FAME (4.27, 5.47); Table S3). Similar results applied to the *n*-3 index.

An increase in the total content of *n*-3 PUFA could only be observed in the MF group, where the difference compared to the HLA and LLA groups was significant. However, the MF group also had lower baseline values than the HLA and LLA groups (*p* < 0.05; Table S3).

Concerning *n*-6 fatty acids, an increase in LA was observed in the HLA, LLA, and control groups by a median of 7–27%, whereas concentrations of C-20:3 (16–22%), ARA (10–16%), C-22:4 (12–30%) and C-22:5 (32–47%), and total *n*-6 PUFA content (4–7%) decreased (*p* < 0.01, exception: HLA group: *n*-6 PUFA: *p* = 0.039; Table S3). The ARA/LA ratio decreased significantly (12–31%) in all groups. Considering the change from baseline, it is noticeable that the increase or reduction in concentrations was more pronounced in the HLA and/or LLA groups compared to the MF and/or control groups.

In the HLA, LLA, and control groups, there was an increase in C-16:0 and a decrease in C-18:0 (*p* < 0.01). C-17:0 only increased in the MF and control groups, and C-18:1c9 and total MUFA only increased in the HLA and LLA groups (*p* < 0.05). An increase in C-14:0 and C-15:0 could only be detected in the control group (*p* < 0.05; Table S3). There were no significant changes in the total SFA concentration during the intervention. The starting values and some final values were higher in the MF and control groups compared to the HLA and LLA groups (*p* < 0.05; Table S3).

The trans-fatty acids (TFA) fell in the HLA and increased in the MF group (*p* < 0.05). The change from baseline in the MF group was significantly different from the HLA and control groups.

### 3.3. Changes in Fatty Acid Distribution in Erythrocyte Lipids per Diet Group Subdivided into Subgroups by Sex, EPA Status, and LA Change over the Intervention Period

To evaluate the conversion rate of ALA into its long-chain metabolites, we examined the influence of the following three influencing factors: sex (men vs. women), EPA status at baseline (<0.9% FAME vs. ≥0.9% FAME), and LA change throughout the study period (<1.5% FAME vs. ≥1.5% FAME). The subgroups mentioned above were formed within the four diet groups (Table 4). The changes within the subgroups are, in principle, comparable to those of the diet groups (Table 4 and Table S3).

A sex-specific difference was only observed for the DPA concentration in the LLA group, which increased significantly in men and remained unchanged in women.

According to EPA status, the subdivision shows that increases in both ETA and EPA were more pronounced at low EPA status than at high EPA status. In contrast, the DHA and *n*-3 index decrease was less pronounced. Statistical significance could be achieved regarding ETA change in the HLA and LLA groups and the EPA change in the MF group (Table 4). The reduction in DHA and *n*-3 index were stronger in the LLA and MF groups of the subgroup with high EPA status (*p* < 0.05; Table 4). The DPA concentration also seemed to increase more in the subgroup with low EPA status compared to the subgroup with high EPA status. However, a statistically significant difference could only be found between the two LLA groups. Regarding the *n*-6 fatty acids under consideration, only a higher increase in LA could be observed in individuals of the HLA group with high EPA status compared to individuals with low EPA status (*p* < 0.05; Table 4). Consequently, the *n*-6/*n*-3 ratio fell more in the HLA, LLA, and MF groups of the subgroup with low EPA status than those with high EPA status (*p* < 0.05; Table 4).

The change in LA concentration throughout the study seems to have less of an impact, as only a few independent significant changes were observed. As expected, the increase in LA differs between the LA change ≥1.5% FAME subgroup and the LA change <1.5% FAME subgroup (*p* < 0.01). This was evident in all diet groups (Table 4). Furthermore, in the subgroup with LA change ≥1.5% FAME, the increase in ALA and DPA in the control group was higher than in the LA change <1.5% FAME subgroup (*p* < 0.05; Table 4). In contrast, in the LA change <1.5% FAME subgroup, ETA and DPA increased more in the LLA group than in the LA change ≥1.5% FAME subgroup (*p* < 0.05; Table 4). In the LA change ≥1.5% FAME subgroup, a greater decrease in DHA concentration as well as *n*-3 index was observed in the LLA group (*p* = 0.007, *p* = 0.012) and a greater decrease in C-22:5n6 concentration in the control group (*p* = 0.012) compared to the LA change <1.5% FAME subgroup (Table 4). On the other hand, the *n*-6/*n*-3 ratio dropped more in the HLA (*p* = 0.046) and LLA groups (*p* = 0.007) of the LA change <1.5% FAME subgroup compared to the LA change ≥1.5% FAME subgroup (Table 4).

Independent of sex or EPA status (EPA baseline status: <0.9 vs. <0.9% FAME), the comparison of diet groups shows a higher increase in DPA and a lower decrease in DHA and the *n*-3 index in the MF group compared to the HLA group (*p* < 0.05; Table 4). The same effects were also found by comparing the EPA subgroups between the diet groups. In contrast, LA increased less and C-22:4n6 and C-22:5n6 decreased less between the HLA and the MF group (*p* < 0.05; Table 4). The *n*-6/*n*-3 ratio also dropped more in the HLA than in the MF group (*p* < 0.05; Table 4). In the comparison between the MF and the LLA and control groups, these differences only partially reached statistical significance. A few other differences between the four groups were found but did not follow a conclusive pattern and are therefore not described in detail.

### 3.4. Influence on Fatty Acid Distribution in Erythrocyte Lipids Depending on Sex, EPA Status, and Total LA Change without the Original Diet Group Split

The intended fatty acid intake provided via the menu plans (Figure 2) was not reflected by the changes in fatty acid distribution in erythrocyte lipids. This indicates that the participants did not regularly follow the menu plan instructions. Because of this, and to increase the sample size, we removed the division into the diet groups to analyze the defined influencing factors (sex differences, EPA status at baseline, total LA change (% FAME) over the study).

**Figure 3:**
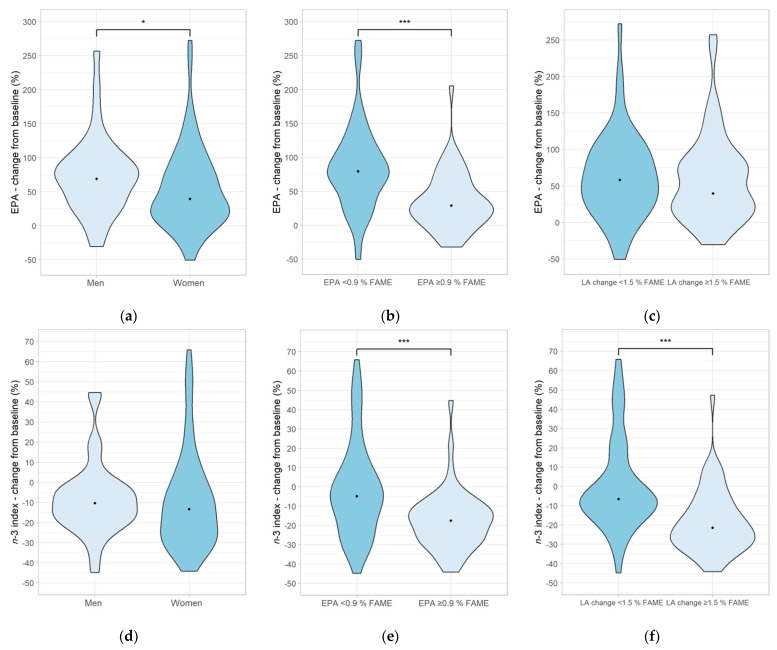
Plots (**a**–**c**) show the percentage change from baseline for EPA in erythrocyte fatty acids. Plots (**d**–**f**) show the percentage change from baseline for the *n*-3 index in erythrocyte fatty acids. Participants were subdivided by sex (men vs. women; plots (**a**,**d**)), by EPA status at baseline (<0.9% FAME vs. ≥0.9% FAME; plots (**b**,**e**)), and by total LA change (% FAME) comparing baseline and the end of the study (<1.5% FAME vs. ≥1.5% FAME; plots (**c**,**f**)). Percentage changes from baseline data stated below (also shown in Table S4) are either expressed as mean (±SD) or as median (25th, 75th percentile) depending on the statistical test that was performed. * *p* < 0.05; *** *p* < 0.001; Plot A: EPA percentage change from baseline, men (69.00 (34.96, 94.35), *n* = 35) vs. women (39.39 (12.85, 79.04), *n* = 68), *p* = 0.043; Plot B: EPA percentage change from baseline, EPA < 0.9% FAME (79.28 (45.72, 111.29), *n* = 54) vs. EPA ≥ 0.9% FAME (28.96 (8.61, 58.01), *n* = 49), *p* < 0.001; Plot C: EPA percentage change from baseline, LA change <1.5% FAME (58.02 (26.25, 98.35), *n* = 52) vs. LA change ≥1.5% FAME (39.40 (16.93, 81.19), *n* = 51); Plot D: *n*-3 index percentage change from baseline, men (−10.41 (−18.16, −2.25), *n* = 35) vs. women (−13.33 (−27.62, 0.32), *n* = 68); Plot E: *n*-3 index percentage change from baseline, EPA < 0.9% FAME (−4.90 (−19.79, 8.21), *n* = 54) vs. EPA ≥ 0.9% FAME (−17.59 (−26.72, −10.40), *n* = 49), *p* < 0.001; Plot F: *n*-3 index percentage change from baseline, LA change <1.5% FAME (−6.53 (−14.16, 7.86), *n* = 52) vs. LA change ≥1.5% FAME (−21.50 (−29.36, −10.41), *n* = 51); *p* < 0.001. Abbreviations: EPA, eicosapentaenoic acid; FAME, fatty acid methyl esters; LA, linoleic acid.

This evaluation shows that in women, EPA increased by a median of 39% (*p* < 0.001), and the *n*-3 index decreased by a median of 13% (*p* < 0.001; Figure 3a,d). In men, a median increase of 69% (*p* < 0.001) and a decrease of 10% (*p* = 0.01) was found, respectively (Figure 3a,d). In contrast to the analysis with the diet group subdivision, an influence of sex was found as men showed a higher conversion rate of ALA to EPA than women (*p* = 0.043; Figure 3a,d).

Subjects with an initial EPA value of <0.9% FAME had a median increase in EPA of 79% (*p* < 0.001) and a decrease in the *n*-3 index of 5% (*p* = 0.1) after 12 weeks. Subjects with an initial EPA value of ≥0.9% FAME showed an increase of 29% (*p* < 0.001) and a decrease of 18% (*p* < 0.001), respectively (Figure 3b,e). Thus, EPA increased more in the EPA < 0.9% FAME subgroup, while the *n*-3 index decreased less compared to the subgroup with EPA ≥ 0.9% FAME (*p* < 0.001). This is similar to the previously described results with the subdicisions into diet groups.

In subjects with LA change <1.5% FAME, median EPA increased by 58% (*p* < 0.001) and the median *n*-3 index decreased by 7% (*p* = 0.212). In subjects with LA change ≥1.5% FAME, the median increase and decrease were 39% (*p* < 0.001) and 22% (*p* < 0.001), respectively. The increase in EPA did not differ significantly depending on LA change over the study period. However, the *n*-3 index decreased less in individuals with LA change <1.5% FAME (*p* = 0.001; Figure 3c,f).

### 3.5. Correlation between Micronutrient Status and EPA Percentage Change from Baseline in the Entire Study Population

A correlation analysis between micronutrient status (calcium, potassium, ferritin, transferrin, folic acid, biotin, holo-transcobalamin, vitamins A, B_1_, B_2_, B_6_, B_12_, C, D, E) at baseline or their change from baseline with the EPA change from baseline was performed on the whole study collective to prove the impact of micronutrient status on ALA conversion into its long-chain metabolites. A correlation between baseline micronutrient status and EPA change from baseline was only found for potassium and vitamin D (*p* < 0.05), which had an effect strength of r = 0.243 and r = 0.209, respectively. The analyses comparing micronutrient changes from baseline with EPA change from baseline only showed a correlation for vitamin D (*p* < 0.05, r = 0.216).

### 3.6. Influence on Biochemical Parameters and Anthropometric Measurements per Diet Group

Regarding the analyzed cardiovascular risk factors (total, LDL, MDA-LDL, high-density lipoprotein (HDL) and non-HDL cholesterol, triglycerides, high-sensitivity CRP, apolipoprotein A1 and B, lipoprotein(a), homocysteine), no significant differences were found between the different groups, both at baseline and at the end of the study (Table 5). However, significant effects were observed for total, LDL, and HDL cholesterol in the changes from baseline. In detail, median total cholesterol was decreased the most within the LLA group (−6.45%), with the difference being significant compared to the LLA (−0.88%) and MF groups (0.99%; Table 5). For the median LDL cholesterol, similar results were observed, with the most pronounced decrease (−5.18%) also in the LLA group, which was stronger than in the HLA (1.95%) and MF groups (2.35%) (*p* < 0.05; Table 5). For median HDL cholesterol, the changes from baseline only differed between the LLA (−5.71%) and MF groups (3.73%) (*p* < 0.05; Table 5). Within the groups, changes in total, LDL, and non-HDL cholesterol were only seen in the LLA group. Here, total cholesterol fell on average by −0.42 mmol/L (*p* < 0.01), median LDL cholesterol by −0.2 mmol/L (*p* < 0.05), and non-HDL cholesterol by −0.36 mmol/L (*p* < 0.001; Table 5). Median high-sensitivity CRP decreased in the MF group (*p* < 0.05). The median ratio of apolipoprotein B to apolipoprotein A increased in all groups, but this was only significant in the HLA (*p* < 0.01) and control groups (*p* < 0.05; Table 5). Apolipoprotein A fell in the HLA, LLA, and control groups (*p* < 0.01; Table 5). Apolipoprotein B fell in the LLA group (*p* < 0.05). Lipoprotein(a) increased in the HLA and LLA groups (*p* < 0.01; Table 5).

For diabetes’ risk markers (fasting blood glucose, fasting insulin, HbA1c), significant changes only were observed within groups. Here, the mean fasting glucose decreased from 5.89 mmol/L to 5.63 mmol/L (*p* < 0.01), 5.6 mmol/L to 5.25 mmol/L (*p* < 0.05) and 5.9 mmol/L to 5.5 mmol/L in the HLA, LLA and control groups, respectively (*p* < 0.05; Table 5). The HbA1c value increased by approx. 0.1% in the control group (*p* < 0.05).

The vitamin E status was not different between and within groups at any point and the change from baseline did also not show any significant differences. Within groups, mean vitamin E status decreased in the LLA and MF groups (*p* < 0.01).

No significant differences were found at baseline or at the end of the study for all anthropometric measures. However, significant changes in BMI were found in the HLA, LLA, and MF groups. The BMI decreased the most in the LLA group by an average of −0.78 kg/m^2^ (*p* < 0.001), followed by the MF group with −0.33 kg/m^2^ (*p* < 0.05). In the HLA group, the median BMI decreased by −0.07 kg/m^2^ (*p* < 0.01). The median waist circumference changed in the HLA (−2.44 cm) and LLA groups (−2.25 cm, *p* < 0.05; Table 5). Body fat decreased in all groups by an average of −1 kg (HLA group, *p* < 0.05), −1.54 kg (LLA group, *p* < 0.001), −1.27 kg (MF group, *p* < 0.05), and −0.99 kg (control group, *p* < 0.05; Table 5).

For systolic and diastolic blood pressure, significant changes only were observed within groups. Here, median systolic and diastolic blood pressure decreased each by −1 mmHG in the HLA group (*p* < 0.05). In the MF group, mean systolic blood pressure decreased by −7.48 mmHG (*p* < 0.05) and diastolic blood pressure by −5.13 mmHg (*p* < 0.01). In the control group, the mean reductions were −7.68 (*p* < 0.01) and −3.11 mmHG (*p* < 0.05). In the LLA group, systolic blood pressure decreased by an almost significant mean of −5.34 mmHg (*p* = 0.057; Table 5).

## 4. Discussion

### 4.1. Effects on Fatty Acid Distribution in Erythrocytes in Response to Linseed Oil Supplementation

An increased amount of EPA and DHA in erythrocytes is associated with reduced cardiovascular risk, which cannot be clearly confirmed for ALA [34,35,36]. Given the increasing numbers of vegetarians and vegans [37] and the generally low intake of EPA and DHA in many countries [16], it is therefore important to investigate whether the conversion of ALA to EPA and DHA can be favorably influenced by variations in LA intake or by the intake of milk fat. In addition, it is of interest to investigate whether sex as well as EPA status influence conversion.

Our study shows that linseed oil supplementation (10 en%, 22–27 mL ≙ 13–16 g ALA) significantly increases ALA, ETA, and EPA concentrations in erythrocytes in all groups. In contrast, the DHA concentration dropped significantly in the HLA, LLA, and control groups. Since DHA is the most prominent fatty acid in erythrocytes in quantity, the *n*-3 index also decreased significantly in these three groups despite the higher percentage increase in EPA. A significant increase in the DPA concentration could only be observed in the MF and control groups. The lack of a significant increase in DPA concentration in the HLA and LLA groups could be due to the higher initial concentrations. Concerning *n*-6 fatty acids, a significant increase in LA was observed in the HLA, LLA, and control groups, whereas the concentrations of metabolites of the *n*-6 metabolism (C-20:3, ARA, C-22:4, C-22:5) decreased.

Greupner and Kutzner et al. (2018) observed a significant increase in ALA, EPA, and DPA concentrations and a significant decrease in C-20:3n6, ARA, C-22:4n6, and DHA concentrations due to a daily intake of 13 g ALA for 12 weeks. In contrast to our study, the LA concentration did not increase [38].

In the study of Wilkinson et al. (2005), a 12-week intervention with 15 g ALA per day led to a significant increase in ALA (225%) and EPA (150%) concentrations. Thus, the ALA increase was weaker, but the EPA increase was more pronounced compared to our study. A significant effect on the DHA concentration could not be determined [39].

Even small amounts of ALA can significantly increase EPA concentration in erythrocytes. In a study by Kuhnt et al. (2016), 5 g of ALA per day for 8 weeks increased ALA, EPA, and DPA concentrations by 201, 32, and 12%, respectively. Again, C-20:3n6 (−5%), C-22:5n6 (−4%), and DHA (−10%) concentrations decreased significantly. LA and ARA did not change significantly [40]. In a study by Barceló-Coblijn et al. (2008), 3.6 g ALA per day for 6 weeks led to a significant increase in ALA and EPA. There were no significant changes in the concentration of DPA, DHA, LA, and ARA [41].

Furthermore, it is described in reviews that ALA supplementation leads to an increase in EPA and, in some studies, also to an increase in DPA concentration, but the DHA concentration mostly remains unchanged or even decreases [22,42,43].

One reason for the unchanged or falling DHA concentration due to ALA supplementation could be that ∆6 desaturase is needed for the desaturation of ALA into stearidonic acid (C-18:4) and is not available for the last step of DHA formation (desaturation of C-24:5n3 to C-24:6n3) at the same time. Both substrates compete for the same enzyme. However, the ∆6 desaturase has a higher affinity for ALA, so less DHA is produced [44].

In addition to *n*-3 metabolism, ∆6 desaturase is also required for *n*-6 metabolism, but with a higher affinity to *n*-3 fatty acids. This may explain the decrease in ARA and C-22:5n6 and the increase in LA [44].

A study on hamsters confirms this assumption. Higher intakes of ALA resulted in lower levels of DHA and ARA in erythrocytes (LA intake unchanged). The LA concentration, however, increased [45].

The characteristic fatty acid intake, which the menu plans should provide, was not reflected by the changes in fatty acid distribution in the erythrocyte lipids.

Differences in fatty acid distribution between the diet groups only rarely occurred. Due to the intended intake of sunflower oil in the HLA and olive oil in the LLA group, LA in the HLA and oleic acid in the LLA group should have increased significantly compared to the other three groups [46,47,48,49]. However, these two fatty acids increased equally in the HLA and LLA groups and only showed a higher increase compared to the MF and control groups. In the MF group, the increase in C-15:0, C-17:0, and TFA should have been different from the other groups due to the intake of dairy products [50,51]. However, this only applied to TFA. Based on these results, we have to conclude that the participants did not regularly implement the menu plans. The partial goal of the study, to investigate the influence of a high vs. low LA intake as well as of milk fat on the conversion of ALA, could therefore not be achieved.

To investigate the influence of sex, EPA status, and the total change in LA throughout the study on ALA conversion, we first observed the influence of these factors in the diet groups. Subsequently, to achieve a larger sample size, the division into the four groups was removed, and an analysis within the subgroups was performed again.

While including the diet groups, no sex-specific difference could be found. However, after dissolving the groups, men showed a significantly higher EPA concentration than women. Previous reviews describe a more efficient conversion of ALA into EPA and DHA in women than in men. The reason for this could be a lower β-oxidation rate (women 22% vs. men 33%; measured over 24 h) of ALA and increased activity of desaturases and elongases due to estrogen [22,42]. In the European Prospective Investigations into Cancer and Nutrition (EPIC) Norfolk Study with almost 5000 women and men, women also showed a more efficient conversion of ALA into *n*-3 LC-PUFA (sum of plasma EPA, DPA, DHA) [52]. One explanation for the contradictory results of our study could be the age of the women at the beginning of the study. They were 57 ± 12 years old on average and therefore were in menopause, where the estrogen level is decreased [53].

The subdivision according to EPA status within diet groups shows that both ETA and EPA increase significantly more in participants with low EPA status (<0.9% FAME) and DHA as well as the *n*-3 index decreasing significantly less than in participants with higher EPA (>0.9% FAME) status at baseline. Thus, it was also evident after the groups were dissolved. These data show that conversion of ALA to EPA is more efficient at low baseline status.

Welch et al. (2010) described a higher conversion of ALA into *n*-3 LC-PUFA (sum of plasma EPA, DPA, DHA) in non-fish-eaters with a lower ALA, EPA, and DHA intake than in fish-eaters with a higher ALA, EPA, and DHA intake. This leads to the difference in LC *n*-3 PUFA status being smaller than expected from the lower intake [52].

In the analysis for changes in LA throughout the study within the diet groups, only isolated differences were found, so no clear statement can be made.

In general, it must be taken into account that only a small sample size was available for some of the calculations, so these results must be critically evaluated.

After removing the diet group subdivision, the *n*-3 index fell significantly less in the LA change <1.5% FAME group compared to the LA change ≥1.5% FAME group. This is due to a significantly lower reduction in DHA, as the difference in EPA increase did not reach statistical significance. As mentioned before, the same enzymes are used to convert ALA and LA into their longer and more unsaturated metabolites. From this, we can conclude that a concomitant high LA intake can weaken the conversion of ALA into EPA and DHA [22]. This could also explain why the *n*-3 index decreased less in the LA change <1.5% FAME subgroup.

In rats and pigs, EPA, DPA, and DHA concentrations in liver lipids or plasma phospholipids were found to be lower at higher LA intakes than at lower LA intakes when ALA intake was unchanged [54,55,56]. In rat erythrocytes, only lower levels of DPA and DHA, but not EPA, were found [57]. The observations in erythrocytes are similar to our results, as in the LA change ≥1.5% FAME subgroup (without division into the HLA and control group), the DHA concentration dropped significantly more than in the LA change <1.5% FAME subgroup, and there was no difference in the EPA concentration between both groups.

Goyens et al. (2006) showed that in healthy men and women, with the same ALA intake (0.4 en%) but different LA intake (7 vs. 3 en%), EPA in plasma phospholipids increased significantly more with lower LA intake. The decrease in DHA concentration did not differ significantly between the two groups [58]. The statistical analyses here accordingly indicate an inverse effect.

Rosell et al. (2005) also confirmed in their study that a high LA intake correlates inversely with the EPA and DHA plasma concentration (% of total fatty acids) [59].

When interpreting the results, however, it should be noted that the actual percentage of ALA metabolized to EPA, DPA, and DHA cannot be determined, as we could not conduct our study with marked, stable isotopes. Thus, it cannot be concluded that the LC *n*-3 PUFA are synthesized directly from the supplied ALA. An unknown proportion of the LC *n*-3 PUFA may have been released or synthesized from the storage fat. In addition, it is also possible that the proportion of ALA metabolized to LC *n*-3 PUFA is higher but is stored in tissues and, therefore, cannot be measured in the blood.

Tracer studies in men have shown that only 6–8% of the ingested ALA is found as EPA, 4–8% as DPA, and 0–4% as DHA in plasma lipids [21,60,61]. In women, the proportion was slightly higher at 21% (EPA), 6% (DPA), and 9% (DHA) [62]. However, in a study with stable isotopes by Pawlosky et al. (2011), which included men and women, the conversion rate was only 0.2% for EPA, 0.13% for DPA, and 0.05% for DHA [63].

In addition to influencing factors like EPA status, sex, or change in LA status, genetic factors, in particular, polymorphisms in the fatty acid desaturase (FADS) and fatty acid elongase (ELOVL) genes, and the transcription factors sterol regulatory element-binding protein-1c (SREBP-1c) and peroxisome proliferator-activated receptor α (PPARα) can also influence the expression of desaturases and elongases and thus the conversion of ALA into LC *n*-3 PUFA [64,65]. Hormones also influence the activity of desaturases. Insulin activates ∆6 desaturase, whereas epinephrine and glucagon inhibit the activity of ∆6 and ∆5 desaturase [66].

Whether micronutrient status influences the activity of desaturases has mainly been investigated in rats, so human studies are still required.

Rat studies indicate that vitamin A supplementation raises the ∆5 and ∆6 desaturase activity (based on product-to-precursor ratios) but decreases the Fads1 (encode ∆5 desaturase) messenger ribonucleic acid (mRNA) concentration [67].

There are contradictory results regarding the influence of folate and vitamin B_12_ on desaturase activity and Fads1 mRNA expression. Wadhwani et al. (2012) showed that high folic acid intake in the absence of vitamin B_12_ caused increased ∆5 desaturase and reduced ∆6 desaturase activities but decreased Fads1 mRNA concentration in rats [67]. In contrast, in a mouse study by Castaño-Moreno et al. (2020), high folic acid and reduced vitamin B_12_ intake decreased the activity of ∆5 and ∆6 desaturase and increased Fads1 mRNA concentration. An effect on Fads2 expression could not be determined in either of the two studies [67].

Iron and zinc are cofactors of desaturases. Therefore, it is plausible that iron and zinc status affect desaturase activities. Zinc deficiency in both rats and humans leads to a decrease in ∆6 desaturase activity. Regarding the ∆5 desaturase activity, rat and human studies show contradictory results. In rats, zinc deficiency leads to a decrease in ∆5 desaturase activity, whereas high serum zinc was associated with low ∆5 desaturase activity in humans. Moreover, iron deficiency impairs LC-PUFA synthesis in rats and humans [67].

### 4.2. Discussion of the Observed Effects on Biochemical and Anthropometric Parameters

In general, effects on cardiovascular risk markers in the intervention groups differ from the literature in terms of adherence to a healthy diet [68,69,70]. In addition to this, the composition of dietary fat recommended by the menu plans was not reflected by changes in fatty acid distribution in erythrocyte lipids. Both results support the hypothesis that the participants did not adequately implement the menu plans. Consequently, only the effect of linseed oil supplementation remains as an influencing factor on cardiovascular risk factors. In principle, positive effects through an increased ALA consumption by itself on cardiovascular and diabetic risk factors are described in the literature, with a range of doses (approx. 1.2–8 g/d ALA) achieving significant effects on, e.g., blood pressure, total cholesterol, LDL cholesterol, triglycerides, insulin, and homeostasis model assessment of insulin resistance (HOMA-IR) with a study duration of 12 weeks [71,72,73]. However, these effects could not be shown consistently in all studies [74,75], and the results of the present study also show an inconsistent picture, as the previously mentioned risk factors were either only slightly or not at all positively influenced. Avelino et al. (2015) investigated the effects of nutritional counselling combined with or without a daily intake of 3 g linseed oil (1.75 g/d ALA) over 12 weeks on blood lipid profiles [71]. For subgroup analysis, both intervention groups were subdivided according to their SFA intake (>7 vs. <7 en% SFA). Here, the reduction in total cholesterol (−0.58 mmol/L vs. −0.80 mmol/L, *p* < 0.020) and LDL cholesterol (−0.72 mmol/L vs. −0.86 mmol/L, *p* < 0.050) was more pronounced among subjects with an SFA intake of >7 en% when linseed oil was also ingested. At the same time, HDL cholesterol increased more among subjects in the linseed oil group than in the other intervention group (0.23 mmol/L vs. 0.04 mmol/L, *p* < 0.001). The data of Avelino et al. (2015) show that a lower amount of linseed oil than in the present study, over a comparable duration, might produce an additive effect that significantly exceeds the effect of nutritional counselling alone [71]. In their intervention study, Paschos et al. (2007) examined the effect of linseed oil supplementation on blood pressure [72]. For 12 weeks, participants received either 8 g ALA (*n* = 59) via linseed oil or 11 g LA via safflower oil daily. Systolic blood pressure was reduced more in the ALA group (−10 mmHG, change from baseline −3.1%) than in the LA group (−5 mmHG, change from baseline −1.7%) (*p* = 0.016). For diastolic blood pressure, the reductions were also different (−8 mmHG, change from baseline −6.3% vs. −1 mmHG, change from baseline −2.5%, *p* = 0.011). These results are comparable to the present study for systolic pressure, as reductions between 2.93% and 4.75% were achieved in all groups. For diastolic blood pressure, however, a comparable result was only reached in the MF group (−5.12%) [72]. In their intervention study, Ristic-Medic et al. (2014) investigated the influence of a seed mixture on various markers such as glycemic control, serum lipids and anthropometric parameters [76]. The subjects consumed ground sesame seeds (6 g), pumpkin seeds (6 g), and linseeds (18 g) daily for 12 weeks (28% ALA in fatty acid profile of the mixture) while maintaining their regular diet. After the intervention, there was a reduction in blood pressure (systolic blood pressure: −10 mmHG, *p* < 0.01; diastolic blood pressure: −6 mmHG, *p* < 0.01), triglycerides (−0.62 mmol/L, change from baseline −30%, *p* < 0.01) and total cholesterol (−0.46 mmol/L, −7%, *p* < 0.05). BMI, HDL, and LDL cholesterol did not change significantly [76]. The results are thus partially comparable with the effects found in the present study since blood pressure (MF and control groups) and total cholesterol (LLA group) were reduced significantly and to a comparable extent. However, the effects were not found in all diet groups and no comparable effect was found for triglycerides in any group. In principle, however, a comparison of the results should be made, considering that a seed mixture contains further beneficial nutrients which can be responsible for the described positive effects on cardiovascular risk factors. Soltani et al. (2013) also used ground seeds in their randomized controlled intervention study, whereby the intervention group received 40 g of linseeds per day [77]. Otherwise, both diet groups continued their regular diet. After 8 weeks, triglycerides (baseline value: 3.31 mmol/L; −1.04 mmol/L), total cholesterol (baseline value: 6.06 mmol/L; −0.91 mmol/L), and LDL cholesterol (baseline value: 3.83 mmol/L; −0.65 mmol/L) were significantly reduced in the intervention group compared to baseline as well as compared to the control group. HDL cholesterol (baseline value 0.96 mmol/L; +0.16 mmol/L), on the other hand, increased over time and in the group comparison (*p* < 0.05) [77]. In this case, more pronounced effects compared to the present study may be related to the intake of further health-promoting nutrients, such as dietary fiber from linseeds.

## 5. Conclusions

Our data show that a daily intake of approx. 25 g linseed oil (≙ approx. 15 g ALA) leads to a significant increase in EPA concentrations and a simultaneous decrease in DHA concentrations in erythrocyte lipids. The increase in EPA was significantly stronger in individuals with a lower EPA status at baseline (<0.9% FAME), highlighting that converting ALA to EPA is more efficient in participants with low EPA. In contrast to most previous findings, we did not find a more efficient conversion of ALA to EPA in women, possibly because our female participants were in menopause. Our data suggest that a high LA status attenuates the conversion of ALA. The micronutrient status seems to have no clear effect on conversion from ALA to EPA or DHA.

The absence of effects on blood lipids, markers of glucose metabolism, and fatty acid distribution in erythrocyte lipids supports the hypothesis of lacking compliance with the menu plans. The fact that no apparent effects on blood lipids and further cardiovascular risk factors were achieved by linseed oil supplementation alone is in line with the literature since effects were not consistently visible.

## 6. Strengths and Limitations

The KoALA study was designed to evaluate influencing factors on the metabolism of land-based *n*-3 PUFA. To examine this influence, detailed menu plans were developed according to three defined nutrient profiles (low LA, high LA, and high in milk fat) and were used as the basis of the intervention. In theory, a diet according to such menu plans allows for precise control of subjects’ nutrient intake. However, implementation of the plans demands a lot of effort and time to execute. Deviations from the plans can therefore be a problem, which was also shown in the present study. The menu plans were calculated with the nutrition software PRODI, which uses the data set of the ‘Bundeslebensmittelschlüssel’ to calculate nutrient profiles for all foods recommended by the plans. However, a calculation based on this database cannot always ensure an actual intake of nutrients according to the intended pattern, as the actual nutrients of the food consumed can only be represented to a limited extent, since, for example, seasonal, regional, and variety-related differences or preparation cannot be taken into account. Another limitation is the particularly small sample size for the calculations on effects within the subgroups before the diet groups were dissolved, so that these results must be interpreted cautiously.

The strengths of the KoALA study are the comprehensive health status assessment focusing on nutrient status and cardiovascular risk factors. Combined with the broad analysis of fatty acids in the erythrocyte lipids, it is possible to picture a variety of influences on *n*-3 PUFA metabolism. In addition, the frequent taking of blood samples throughout the study enables the evaluation of observed effects over the time periods. Compliance with the intended intake of linseed oil was enhanced by providing it to each participant as part of the study.

## Figures and Tables

**Table 1:** Fatty acid distribution of the provided linseed oil expressed as fatty acid methyl esters (FAME).

Fatty Acid Distribution of Linseed Oil (% FAME)							
C-16:0	C-18:0	C-18:1c9	C-18:2c9, c12	aC-18:3c9, c12, c15	SFA	MUFA	PUFA
4.6	2.9	17.5	13.7	60.2	7.8	18.3	74.0

Abbreviations: MUFA, monounsaturated fatty acids; FAME, fatty acid methyl esters; PUFA, polyunsaturated fatty acids; SFA, saturated fatty acids.

**Table 2:** Characteristics of the study collective—baseline assessment.

Characteristics of the Study Collective 69 w (65.7%); 36 m (34.3%)								
Age (Years)	BMI (kg/m^2^)	Total Cholesterol (mmol/L)	LDL Cholesterol (mmol/L)	HDL Cholesterol (mmol/L)	Triglycerides (mmol/L)	Lipoprotein(a) (mg/L)	Systolic Blood Pressure (mmHG)	Diastolic Blood Pressure (mmHG)
57.4 (±12.1)	25.6 (23.4, 29.1)	5.8 (±1.2)	3.6 (±1.0)	1.6 (±1.0)	1.1 (0.9, 1.5)	88.5 (40.3, 264.5)	140.5 (±21.1)	86.9 (±11.0)

Variables expressed as mean (±SD) or as median (25th, 75th percentile) depending on the data distribution. Abbreviations: BMI, body mass index; HDL, high-density lipoprotein; LDL, low-density lipoprotein.

**Table 3:** Sex distribution and age per group—baseline assessment.

	High Linoleic Acid 15 w (55.6%) 12 m (44.4%)		Low Linoleic Acid 19 w (70.4%) 8 m (29.6%)		High Milk 17 w (73.9%) 6 m (26.1%)		Control 18 w (65.7%) 10 m (34.3%)	
Age (years)	57.7 (±11.5)	a	56.8 (±11.6)	a	55.8 (±12.3)	a	58.9 (±13.4)	a

Variable expressed as mean (±SD) and/or as median (25th, 75th percentile) depending on the statistical test that was performed; groups without a common letter are significantly different, *p* < 0.05.

**Table 4:** Comparison of percentage change from baseline within diet groups split in dependence of sex, EPA baseline status, and LA change over the intervention period.

Subgroups	High Linoleic Acid				Low Linoleic Acid				High Milk				Control			
	Cfb (%) *	∆	◊	*n*	Cfb (%) *	∆	◊	*n*	Cfb (%) *	∆	◊	*n*	Cfb (%) *	∆	◊	*n*
aC-18:3c9c12c15 (ALA)																
Women	440.07 (±235.72)		a	15	355.28 (±246.75)		a	19	389.05 (±267.22)		a	17	300.40 (±174.32)		a	17
Men	496.34 (±349.31)	n.s.	a	12	352.12 (±171.60)	n.s.	a	8	341.87 (±207.12)	n.s.	a	5	288.55 (±132.89)	n.s.	a	9
EPA (<0.9)^Baseline^	463.30 (±264.72) 511.27 (206.41, 700.75)		a	14	310.82 (±217.39)		a	13	407.49 (±245.47)		a	14	295.96 (±136.85)		a	12
EPA (≥0.9)^Baseline^	350.23 (263.60, 557.27)	n.s.	a	13	381.07 (±233.81) 404.86 (179.94, 579.31)	n.s.	a	14	350.20 (±268.10) 395.00 (112.72, 495.26)	n.s.	a	8	278.03 (±173.12) 207.75 (166.42, 364.09)	n.s.	a	13
LA (<1.5)^Change^	512.00 (±271.13) 532.77 (424.35, 620.47)		a	4	301.76 (±198.13) 386.47 (119.93, 464.86)		a	7	354.17 (±227.09) 343.57 (162.18, 524.13)		a	20	261.63 (±164.96) 265.22 (187.96, 324.90)		a	20
LA (≥1.5)^Change^	399.88 (254.99, 699.00)	n.s.	a	23	429.40 (242.02, 562.22)	n.s	a	20	619.88 (456.14, 783.63)	n.s	a	2	446.61 (288.99, 464.50)	0.037	a	6
C-20:4n3 (ETA)																
Women	81.12 (±110.66)		a,b	15	60.27 (±87.56)		a	19	156.49 (±134.89)		b	17	92.94 (±61.36)		a,b	14
Men	108.16 (±97.61)	n.s.	a	12	113.86 (±68.37)	n.s.	a	8	188.56 (±63.19)	n.s.	a	5	100.29 (±95.39)	n.s.	a	8
EPA (<0.9)^Baseline^	135.20 (±104.60) 138.04 (61.09, 225.49)		a	14	114.61 (±89.01)		a	13	182.42 (±133.39)		a	14	91.13 (±55.75)		a	8
EPA (≥0.9)^Baseline^	30.02 (−6.89, 80.63)	0.025	a	13	33.65 (±57.69) 28.81 (−11.25, 65.11)	0.011	a	14	131.16 (±97.40) 120.49 (65.12, 205.27)	n.s.	b	8	109.17 (±83.16) 83.58 (53.36, 129.30)	n.s.	b	12
LA (<1.5)^Change^	137.34 (±78.69) 117.32 (78.86, 175.80)		a	4	135.06 (±100.98) 165.04 (43.66, 202.61)		a	7	154.90 (±112.80) 140.21 (92.79, 205.27)		a	20	57.78 (±52.16) 62.71 (27.46, 82.67)		a	18
LA (≥1.5)^Change^	47.17 (7.69, 142.03)	n.s	a	23	37.90 (9.27, 107.54)	0.030	a	20	252.59 (171.98, 333.19)	n.s	a	2	115.20 (85.39, 141.34)	n.s	a	4
C-20:5n3 (EPA)																
Women	59.81 (24.86, 76.67)		a	15	51.62 (±49.10) 28.13 (16.47, 84.55)		a	19	68.98 (±75.86) 58.03 (13.06, 108.47)		a	17	32.47 (±37.81) 29.83 (8.61, 42.52)		a	17
Men	79.40 (±76.71) 73.35 (29.75, 101.59)	n.s.	a	12	64.33 (±26.56)	n.s.	a	8	92.70 (±25.56)	n.s.	a	5	66.83 (±64.39)	n.s.	a	10
EPA (<0.9)^Baseline^	107.50 (±78.73)		a	14	74.00 (±41.07)		a	13	98.73 (±68.92)		a	14	56.71 (±45.75) 68.44 (23.32, 101.10)		a	13
EPA (≥0.9)^Baseline^	32.10 (±35.28) 28.96 (−3.47, 68.84)	0.004	a	13	38.53 (±39.89) 22.22 (14.61, 68.02)	0.032	a	14	34.02 (±43.98) 26.22 (8.96, 59.44)	0.027	a	8	32.43 (11.06, 39.89)	n.s.	a	14
LA (<1.5)^Change^	117.27 (±105.52) 79.58 (61.35, 135.50)		a	4	67.79 (±32.73) 75.83 (47.03, 87.31)		a	7	65.75 (±59.93) 60.72 (27.77, 106.44)		a	20	30.92 (±44.66) 29.79 (4.67, 53.14)		a	21
LA (≥1.5)^Change^	59.81 (24.86, 79.28)	n.s	a	23	50.25 (15.69, 81.12)	n.s	a	20	160.59 (121.44, 199.75)	n.s	a	2	26.57 (19.64, 35.34)	n.s	a	6
C-22:5n3 (DPA)																
Women	−3.02 (±26.18)		a	15	−4.82 (±23.00)		a	19	34.26 (±49.65)		b	17	8.89 (±15.43)		a	17
Men	−1.40 (±19.34)	n.s.	a	12	17.56 (±16.58)	0.020	b	8	41.11 (±11.51)	n.s.	c	5	15.31 (±13.64)	n.s.	b	10
EPA (<0.9)^Baseline^	2.68 (±26.35)		a	14	11.77 (±20.35)		a,b	13	40.90 (±51.54)		b	14	13.75 (±16.20)		a,b	13
EPA (≥0.9)^Baseline^	−7.66 (±18.21)	n.s.	a	13	−7.55 (±22.80)	0.029	a	14	28.32 (±29.17)	n.s.	b	8	8.97 (±13.69)	n.s.	a,b	14
LA (<1.5)^Change^	13.94 (±35.11) 17.52 (−4.90, 36.36)		a	4	17.54 (±19.96) 24.32 (12.17, 29.05)		a	7	33.73 (±43.23) 35.27 (8.32, 48.14)		a	20	8.69 (±14.26) 8.74 (−4.03, 20.06)		a	21
LA (≥1.5)^Change^	−12.91 (−16.82, 12.83)	n.s	a	23	−6.99 (−15.93, 6.69)	0.036	a	20	56.72 (35.29, 78.16)	n.s	a,b	2	17.39 (10.91, 33.93)	0.026	b	6
C-22:6n3 (DHA)																
Women	−27.60 (±19.16)		a	15	−32.59 (±14.65)		a	19	2.80 (±32.21)		b	17	−20.72 (±13.63)		a	17
Men	−32.70 (±12.76)	n.s.	a	12	−26.88 (±9.38)	n.s.	a,b	8	−4.30 (±24.92)	n.s.	c	5	−15.31 (±11.67)	n.s.	b,c	10
EPA (<0.9)^Baseline^	−25.38 (±19.61)		a	14	−24.23 (±13.19)		a	13	11.94 (±31.47)		b	14	−20.32 (±13.72)		a	13
EPA (≥0.9)^Baseline^	−34.70 (±11.27)	n.s.	a	13	−36.77 (±11.06)	0.013	a	14	−18.63 (±14.67)	0.018	b	8	−17.23 (±12.58)	n.s.	b	14
LA (<1.5)^Change^	−10.49 (−34.11, 8.57)		a	4	−17.25 (−23.05, −14.31)		a	7	−7.01 (−22.77, 17.87)		a	20	−14.00 (−23.35, −11.72)		a	21
LA (≥1.5)^Change^	−34.10 (−41.68, −20.77)	n.s	a	23	−37.03 (−40.61, −29.90)	0.007	a	20	5.88 (1.37, 10.39)	n.s	a	2	−21.70 (−35.15, −12.34)	n.s	a	6
*n*-3 index																
Women	−25.95 (−28.58, −9.96)		a	15	−20.49 (±15.70) −23.91 (−32.02, −6.83)		a	19	9.81 (±27.95) −0.04 (−9.27, 29.80)		b	17	−14.12 (−21.15, −3.20)		a,b	17
Men	−18.16 (−23.46, −13.09)	n.s.	a	12	−12.05 (±8.78) −12.23 (−16.09, −6.14)	n.s.	a,b	8	11.82 (±22.98) 18.58 (−6.18, 18.77)	n.s.	b	5	−4.70 (−9.67, −2.17)	n.s.	a,b	10
EPA (<0.9)^Baseline^	−9.17 (±26.00)		a	14	−9.96 (±13.12)		a	13	21.34 (±25.26)		b	14	−9.34 (±16.90) −5.73 (−28.66, 1.94)		a	13
EPA (≥0.9)^Baseline^	−23.89 (±9.93) −25.95 (−28.85, −16.77)	n.s.	a	13	−25.34 (±11.58) −26.46 (−32.31, −21.42)	0.003	a	14	−9.38 (±14.49) −11.42 (−15.40, −6.71)	0.002	b	8	−12.20 (−16.88, −4.22)	n.s.	b	14
LA (<1.5)^Change^	4.48 (±45.02) 3.27 (−25.29, 33.03)		a	4	−6.67 (±10.24) −5.72 (−11.24, −1.16)		a	7	8.53 (±26.35) −3.11 (−9.58, 25.31)		a	20	−9.58 (±13.30) −9.61 (−13.93, −0.63)		a	21
LA (≥1.5)^Change^	−20.12 (−28.56, −13.27)	n.s	a	23	−24.01 (−31.72, −13.14)	0.012	a	20	27.61 (17.80, 37.41)	n.s	a	2	−18.65 (−28.86, −5.49)	n.s	a	6
C-18:2c9c12 (LA)																
Women	28.30 (±11.89) 29.78 (23.63, 35.54)		a	15	20.70 (±13.85) 21.83 (12.02, 31.19)		a	19	4.19 (−3.48, 6.35)		b	17	6.14 (−0.14, 10.64)		b	17
Men	26.12 (±8.28) 26.92 (20.88, 31.42)	n.s.	a	12	20.44 (±7.25) 20.29 (16.21, 23.04)	n.s.	a,b	8	9.54 (4.54, 9.81)	n.s.	c	5	9.13 (5.51, 13.36)	n.s.	b,c	10
EPA (<0.9)^Baseline^	23.00 (±10.56) 23.64 (14.28, 28.44)		a	14	15.95 (±12.08) 15.53 (10.80, 21.73)		a,b	13	5.55 (2.56, 8.83)		c	14	9.50 (5.40, 14.12)		b,c	13
EPA (≥0.9)^Baseline^	32.00 (±8.02) 31.91 (27.30, 36.31)	0.020	a	13	24.96 (±10.82) 23.87 (18.37, 30.90)	n.s.	a	14	3.39 (−1.00, 6.43)	n.s.	b	8	5.98 (−2.99, 9.48)	n.s.	b	14
LA (<1.5)^Change^	10.16 (8.76, 11.71)		a	4	9.31 (2.39, 10.47)		a	7	4.36 (−2.43, 6.44)		a	20	5.52 (−0.14, 8.76)		a	21
LA (≥1.5)^Change^	29.78 (24.88, 34.85)	<0.001	a	23	23.87 (18.44, 32.60)	<0.001	a	20	20.66 (17.36, 23.96)	0.009	a	2	21.65 (19.07, 26.39)	<0.001	a	6
C-20:4c5c8c11c14 (ARA)																
Women	−14.69 (±7.57) −16.22 (−21.35, −10.14)		a	15	−12.82 (±8.43) −12.92 (−18.09, −6.52)		a	19	−7.08 (−14.83, 6.22)		a	17	−14.26 (±7.52) −14.16 (−19.13, −7.68)		a	17
Men	−13.90 (±8.32)	n.s.	a	12	−9.43 (±7.14)	n.s.	a	8	−1.31 (±4.31) −2.33 (−2.51, 0.50)	n.s.	a	5	−13.22 (±19.52)	n.s.	a	10
EPA (<0.9)^Baseline^	−16.43 (±7.51) −17.43 (−20.90, −12.03)		a	14	−10.85 (±7.71) −10.01 (−16.64, −6.36)		a,b	13	−4.64 (−11.00, 5.92)		b	14	−13.43 (±5.44) −13.52 (−16.75, −11.18)		a,b	13
EPA (≥0.9)^Baseline^	−12.10 (±7.67)	n.s.	a	13	−12.67 (±8.62)	n.s.	a	14	−7.40 (±9.07) −4.80 (−14.65, −1.76)	n.s.	a	8	−14.28 (±17.50)	n.s.	a	14
LA (<1.5)^Change^	−16.58 (−18.54, −12.14)		a	4	−6.53 (−9.43, −5.95)		a	7	−4.80 (−13.84, 4.81)		a	20	−13.52 (−16.12, −9.42)		a	21
LA (≥1.5)^Change^	−13.99 (−20.20, −9.14)	n.s	a	23	−14.49 (−18.03, −9.29)	n.s	a	20	0.17 (−5.15, 5.48)	n.s	a	2	−16.06 (−21.75, −8.66)	n.s	a	6
C-22:4n6																
Women	−27.13 (±14.12)		a	15	−25.63 (±16.45)		a	19	−3.74 (±25.66)		b	17	−11.18 (±14.77)		b	17
Men	−27.72 (±18.55)	n.s.	a	12	−12.71 (13.33)	n.s.	a,b	8	0.62 (±7.60)	n.s.	b	5	−13.71 (±15.47)	n.s.	a,b	10
EPA (<0.9)^Baseline^	−29.03 (±15.90)		a	14	−15.83 (±13.95)		a,b	13	−4.33 (±23.23) −1.97 (−20.59, 12.07)		b	14	−10.07 (±7.66)		b	13
EPA (≥0.9)^Baseline^	−25.63 (±16.36) −30.21 (−37.30, −14.59)	n.s.	a	13	−27.39 (±17.12) −31.58 (−40.73, −17.35)	n.s.	a	14	2.95 (±30.14) −3.21 (−18.16, 7.33)	n.s.	b	8	−14.01 (±19.36) −22.27 (−12.38, 0.63)	n.s.	a,b	14
LA (<1.5)^Change^	−38.80 (−70.56, −23.11)		a	4	−12.17 (−14.32, −4.42)		a	7	−2.00 (−24.30, 12.16)		a	20	−13.96 (−22.16, −4.27)		a	21
LA (≥1.5)^Change^	−43.28 (−63.97, −15.25)	n.s	a	23	−35.16 (−59.88, −20.40)	n.s	a	20	1.55 (−1.35, 4.44)	n.s	a	2	−3.30 (−63.92, −1.54)	n.s	a	6
C-22:5n6																
Women	−43.98 (±13.90) −48.41 (−54.91, −37.67)		a	15	−42.01 (±17.11)		a	19	−16.81 (±29.00)		b	17	−29.37 (±21.93)		a,b	17
Men	−45.43 (−59.05, −37.93)	n.s.	a	12	−32.65 (±14.02) −36.72 (−38.57, −28.21)	n.s.	a,b	8	−13.14 (±6.21) −10.99 (−14.46, −10.00)	n.s.	b	5	−26.06 (±33.16) −30.96 (−46.41, −21.32)	n.s.	a,b	10
EPA (<0.9)^Baseline^	−46.52 (±14.72) −49.12 (−59.09, −35.55)		a	14	−32.92 (±14.41)		a,b	13	−18.83 (±28.62) −31.14 (−14.37, −0.65)		b	14	−35.75 (±19.14)		a,b	13
EPA (≥0.9)^Baseline^	−43.48 (−54.67, −39.87)	n.s.	a,b	13	−44.21 (±17.51) −46.05 (−56.95, −38.57)	n.s.	a	14	−16.59 (−24.20, 0.53)	n.s.	c	8	−21.09 (±30.15) −32.05 (−40.23, −1.28)	n.s.	b,c	14
LA (<1.5)^Change^	−45.96 (−52.19, −35.88)		a	4	−28.67 (−34.70, −21.42)		a	7	−12.72 (−26.63, 1.94)		a	20	−29.23 (−38.88, −15.85)		a	21
LA (≥1.5)^Change^	−46.72 (−56.48, −36.62)	n.s	a	23	−43.60 (−52.52, −38.01)	n.s.	a	20	−23.46 (−28.04, −18.87)	n.s	a	2	−59.63 (−65.10, −44.86)	0.012	a	6
*n*-6/*n*-3																
Women	5.90 (±23.85)		a,b	15	7.70 (±19.78)		a	19	−20.72 (±15.89)		c	17	−5.98 (±14.48) −6.19 (−17.84, 3.56)		b	17
Men	2.87 (±16.60) 1.79 (−11.20, 15.65)	n.s.	a	12	−4.47 (±9.50) −5.35 (−7.04, −1.29)	n.s.	a,b	8	−20.38 (±9.71) −23.95 (−27.34, −15.85)	n.s.	b	5	−10.87 (−16.16, −7.78)	n.s.	a,b	10
EPA (<0.9)^Baseline^	−4.13 (±20.01)		a	14	−5.38 (±14.45)		a	13	−27.40 (±12.05)		b	14	−7.04 (±15.07)		a	13
EPA (≥0.9)^Baseline^	13.90 (±17.46)	0.020	a	13	12.88 (±17.00)	0.006	a	14	−9.30 (±11.52)	0.003	b	8	−10.18 (±12.51)	n.s.	b	14
LA (<1.5)^Change^	−14.27 (±26.68) −13.91 (−31.32, 3.15)		a	4	−11.21 (±10.68) −11.00 (−17.77, −4.97)		a	7	−20.09 (±14.73) −16.59 (−29.14, 11.63)		a	20	−7.77 (±15.27) −12.26 (−17.38, −1.12)		a	21
LA (≥1.5)^Change^	6.63 (−4.99, 18.75)	0.046	a	23	8.21 (−3.05, 19.21)	0.007	a	20	−26.11 (−31.47, −20.75)	n.s	a	2	−3.55 (−10.08, 1.28)	n.s	a	6

* Variables expressed as mean (±SD) and/or as median (25th, 75th percentile) depending on the statistical tests that were performed; ∆ comparison of subgroups within diet groups; ◊ comparison of subgroups between diet groups, groups without a common letter are significantly different, *p* < 0.05; ^Baseline^ subgroup built by using baseline EPA status (<0.9% FAME vs. ≥0.9% FAME); ^Change^ subgroup built by using total LA change (% FAME) that was observed throughout the study (<1.5% FAME vs. ≥1.5% FAME). Abbreviations: ARA, arachidonic acid; Cfb (%), percentage change from baseline; DHA, docosahexaenoic acid; DPA, docosapentaenoic acid; EPA, eicosapentaenoic acid; ETA, eicosatetraenoic acid; LA, linoleic acid.

**Table 5:** Fasting biochemical parameters and anthropometric measurements at baseline (week 0) and at the end of the intervention period (week 12).

Biochemical Parameters and Anthropometric Measurements		Week	High Linoleic Acid			Low Linoleic Acid			High Milk			Control		
			Characteristics *	*p* ^∆^	◊	Characteristics *	*p* ^∆^	◊	Characteristics *	*p* ^∆^	◊	Characteristics *	*p* ^∆^	◊
Total cholesterol (mmol/L) (*n* ^†^ = 26, 27, 23, 28)		0	5.70 (±1.36)		a	6.07 (±0.94)		a	5.84 (±1.24)		a	5.76 (±1.07)		a
		12	5.65 (±0.93)	n.s.	a	5.65 (±1.06)	0.001	a	5.84 (±1.27)	n.s.	a	5.65 (±1.14)	n.s.	a
	Cfb (%)		−0.88 (−8.42, 5.37)		a	−6.45 (−13.68, −2.57)		b	0.99 (−3.97, 6.34)		a	−3.73 (−7.44, 3.24)		a,b
LDL cholesterol (mmol/L) (*n* ^†^ = 26, 27, 23, 28)		0	3.57 (±1.07)		a	3.77 (±0.73)		a	3.58 (±1.12)		a	3.50 (±0.89)		a
		12	3.65 (±0.76)	n.s.	a	3.57 (±0.86)	0.029	a	3.67 (±1.15)	n.s.	a	3.52 (±0.96)	n.s.	a
	Cfb (%)		1.95 (−7.19, 12.4)		a	−5.18 (−13.62, −0.85)		b	2.35 (−6.20, 12.82)		a	−1.67 (−7.73, 6.61)		a,b
MDA-LDL cholesterol (U/L) (*n* ^†^ = 27, 27, 23, 28)		0	67.20 (57.60, 92.75)		a	68.00 (55.60, 106.50)		a	72.24 (±22.35) 70.20 (60.15. 90.30)		a	72.75 (±20.82) 70.20 (60.70, 85.28)		a
		12	80.91 (±30.78) 77.30 (64.25, 97.55)	n.s.	a	70.78 (±22.26) 67.70 (56.50, 86.60)	n.s.	a	73.38 (±30.77)	n.s.	a	76.22 (±32.57)	n.s.	a
	Cfb (%)		2.09 (−26.68, 39.31)		a	−15.60 (−25.04, 6.29)		a	9.46 (−14.67, 15.52)		a	2.66 (−13.30, 23.54)		a
HDL cholesterol (mmol/L) (*n* ^†^ = 26, 27, 23, 28)		0	1.50 (±0.33) 1.52 (1.23, 1.75)		a	1.62 (±0.35) 1.55 (1.41, 1.88)		a	1.56 (1.40, 1.70)		a	1.62 (±0.34) 1.59 (1.42, 1.83)		a
		12	1.46 (±0.32)	n.s.	a	1.56 (±0.32)	n.s.	a	1.65 (±0.43) 1.57 (1.38, 1.73)	n.s.	a	1.60 (±0.35)	n.s.	a
	Cfb (%)		−4.07 (−5.88, 4.04)		a,b	−5.71 (−10.56, 2.31)		a	3.73 (−1.67, 7.60)		b	−0.55 (−5.91, 3.22)		a,b
Non-HDL cholesterol (mmol/L) (*n* ^†^ = 26, 27, 23, 28)		0	4.20 (±1.24)		a	4.45 (±0.92)		a	4.23 (±1.22)		a	4.14 (±1.04)		a
		12	4.18 (±0.86)	n.s.	a	4.09 (±1.04)	0.001	a	4.19 (1.27)	n.s.	a	4.05 (±1.09)	n.s.	a
	Cfb (%)		−0.83 (−8.97, 6.89)		a	−7.59 (−14.63, −3.69)		a	0.32 (−6.70, 6.76)		a	−4.47 (−9.80, 4.83)		a
LDL/HDL (mmol/L) (*n* ^†^ = 26, 27, 23, 28)		0	2.46 (±0.76) 2.57 (1.94, 2.71)		a	2.30 (1.86, 2.79)		a	2.40 (±1.02) 2.17 (1.65, 3.27)		a	2.26 (±0.76) 2.13 (1.72, 2.71)		a
		12	2.61 (±0.77) 2.46 (1.97, 3.23)	n.s.	a	2.20 (1.81,2.74)	n.s.	a	2.39 (±1.03) 2.19 (1.56, 3.21)	n.s.	a	2.31 (±0.84) 2.34 (1.57, 2.83)	n.s.	a
	Cfb (%)		3.63 (−4.06, 11.25)		a	−2.66 (−6.38, 5.12)		a	−1.33 (−9.21, 6.98)		a	−0.01 (−7.95, 11.95)		a
Triglycerides (mmol/L) (*n* ^†^ = 26, 27, 23, 28)		0	1.21 (1.01, 1.33)		a	1.09 (0.92, 1.67)		a	0.93 (0.77, 1.18)		a	1.00 (0.90, 1.36)		a
		12	1.27 (1.07, 1.67)	n.s.	a	1.01 (0.90, 1.63)	n.s.	a	1.19 (0.88, 1.35)	n.s.	a	1.16 (0.79, 1.46)	n.s.	a
	Cfb (%)		12.71 (−13.13, 34.61)		a	−5.85 (−14.95, 15.83)		a	14.02 (−5.58, 36.85)		a	1.63 (−16.68, 42.64)		a
High-sensitivity CRP (mg/L) (*n* ^†^ = 26, 27, 23, 28)		0	1.20 (0.60, 1.90)		a	1.10 (0.75, 2.70)		a	1.70 (1.00, 2.90)		a	0.85 (0.40, 1.93)		a
		12	1.35 (0.50, 2.58)	n.s.	a	0.90 (0.40, 2.30)	n.s.	a	1.30 (0.65, 2.05)	0.031	a	0.85 (0.40, 1.93)	n.s.	a
	Cfb (%)		−1.79 (−24.74, 0.00)		a	−16.67 (−36.04, 2.50)		a	−21.43 (−50.00, 12.04)		a	0.00 (−25.75, 20.36)		a
Apolipoprotein B/apolipoprotein A1 (*n* ^†^ = 27, 27, 23, 28)		0	0.71 (±0.21) 0.71 (0.58, 0.77)		a	0.64 (0.55, 0.80)		a	0.71 (±0.26) 0.63 (0.50, 0.90)		a	0.66 (±0.18) 0.64 (0.55, 0.76)		a
		12	0.78 (±0.23) 0.75 (0.63, 1.00)	0.004	a	0.66 (0.53, 0.80)	n.s.	a	0.72 (±0.27) 0.66 (0.52, 0.91)	n.s.	a	0.70 (±0.21) 0.68 (0.56, 0.83)	0.022	a
	Cfb (%)		7.61 (2.78, 12.59)		a	2.00 (−6.64, 9.05)		a	1.59 (−5.88, 10.40)		a	1.95 (−1.17, 10.01)		a
Apolipoprotein A1 (g/L) (*n* ^†^ = 27, 27, 23, 28)		0	1.65 (±0.28)		a	1.72 (±0.26)		a	1.66 (±0.32) 1.66 (1.49, 1.81)		a	1.68 (±0.24)		a
		12	1.53 (±0.29) 1.47 (1.33, 1.76)	<0.001	a	1.60 (±0.25) 1.53 (1.47, 1.77)	0.001	a	1.60 (1.38, 1.79)	n.s.	a	1.60 (±0.25) 1.57 (1.46, 1.68)	0.001	a
	Cfb (%)		−7.33 (±6.34)		a	−6.61 (±9.10)		a	−1.84 (±8.37)		a	−4.89 (±6.70)		a
Apolipoprotein B (g/L) (*n* ^†^ = 27, 27, 23, 28)		0	1.04 (0.94, 1.27)		a	1.18 (±0.27) 1.14 (0.97, 1.35)		a	1.13 (±0.31) 1.08 (0.86, 1.32)		a	1.10 (±0.26) 1.14 (0.90, 1.25)		a
		12	1.15 (±0.25) 1.12 (0.95, 1.37)	n.s.	a	1.11 (±0.29)	0.027	a	1.13 (±0.31)	n.s.	a	1.09 (±0.28)	n.s.	a
	Cfb (%)		−0.96 (−7.08, 6.72)		a	−6.11 (−11.80, 0.00)		a	0.00 (−6.35, 6.40)		a	−0.91 (−8.14, 3.62)		a
Lipoprotein(a) (mg/L) (*n* ^†^ = 26, 27, 23, 28)		0	109.50 (51.00, 304.00)		a	65.00 (30.50, 263.50)		a	87.00 (51.00, 265.50)		a	86.00 (43.00, 206.25)		a
		12	116.50 (64.00, 351.50)	0.002	a	67.00 (45.50, 325.50)	0.002	a	92.00 (50.00, 288.00)	n.s.	a	116.50 (55.75, 234.00)	n.s.	a
	Cfb (%)		16.21 (−1.76, 35.35)		a	19.05 (5.25, 35.37)		a	7.48 (−1.52, 23.40)		a	11.53 (−6.80, 27.04)		a
Homocysteine (µmol/L) (*n* ^†^ = 27, 27, 23, 28)		0	10.70 (8.65, 12.80)		a	9.45 (±1.99) 9.90 (7.70, 10.40)		a	8.70 (8.15, 9.90)		a	9.30 (7.87, 11.93)		a
		12	9.70 (8.35, 11.30)	n.s.	a	10.06 (±2.17) 10.00 (8.90, 11.45)	n.s.	a	9.50 (7.55, 11.50)	n.s.	a	9.80 (7.85, 11.63)	n.s.	a
	Cfb (%)		−4.04 (−24.30, 8.78)		a	10.19 (−6.31, 23.77)		a	6.10 (−19.16, 27.13)		a	1.61 (−12.42, 13.71)		a
Fasting blood glucose (mmol/L) (*n* ^†^ = 27, 26, 23, 28)		0	5.89 (±0.47) 5.90 (5.60, 6.20)		a	5.60 (5.33, 6.23)		a	5.50 (5.20, 5.75)		a	5.09 (5.40, 6.10)		a
		12	5.63 (±0.56) 5.60 (5.35, 5.75)	0.006	a	5.25 (5.10, 5.73)	0.018	a	5.40 (5.05, 5,80)	n.s.	a	5.50 (5.30, 5.90)	0.027	a
	Cfb (%)		−3.85 (−9.92, 0.76)		a	−3.44 (−7.99, 0.00)		a	−1.82 (−7.35, 4.12)		a	−3.39 (−7.42, 1.83)		a
HbA1c (%) (*n* ^†^ = 27, 27, 23, 28)		0	5.31 (±0.36) 5.30 (5.15, 5.50)		a	5.30 (5.00, 5.50)		a	5.30 (5.10, 5.55)		a	5.30 (5.08, 5.50)		a
		12	5.36 (±0.29) 5.40 (5.20, 5.60)	n.s.	a	5.20 (5.05, 5.55)	n.s.	a	5.40 (5.20, 5.50)	n.s.	a	5.40 (5.18, 5.50)	0.015	a
	Cfb (%)		1.82 (−1.84, 3.77)		a	1.85 (−1.92, 2.63)		a	0.00 (−1.75, 3.89)		a	1.62 (0.00, 3.67)		a
Fasting insulin (mU/L) (*n* ^†^ = 26, 27, 23, 27)		0	8.70 (±3.42) 8.10 (6.05, 11.43)		a	7.50 (4.60, 9.55)		a	6.30 (5.35, 9.65)		a	6.20 (4.95, 7.80)		a
		12	8.67 (±3.85) 7.95 (6.00, 11.25)	n.s.	a	6.80 (4.35, 8.00)	n.s.	a	5.70 (4.30, 8.50)	n.s.	a	6.00 (4.85, 7.60)	n.s.	a
	Cfb (%)		−9.94 (−29.53, 24.51)		a	−12.50 (−32.32, 8.52)		a	−13.83 (−30.59, 5.24)		a	−6.49 (−21.05, 8.72)		a
Vitamin E (mg) (*n* ^†^ = 27, 27, 23, 28)		0	34.13 (±8.37)		a	36.57 (±6.32)		a	37.05 (±9.04)		a	35.59 (±7.34)		a
		12	33.60 (±6.78)	n.s.	a	32.91 (±7.35)	<0.001	a	33.58 (±9.08)	0.004	a	33.58 (±6.80)	n.s.	a
	Cfb (%)		−5.47 (−16.03, 11.25)		a	−14.33 (−16.85, −5.60)		a	−7.14 (−14.58, −3.59)		a	−3.86 (−14.65, 4.51)		a
Body weight (kg) (*n* ^†^ = 27, 27, 23, 28)		0	77.86 (±12.06)		a	80.11 (±14.73)		a	75.57 (±16.41)		a	73.44 (±14.44)		a
		12	76.80 (±11.69)	0.013	a	77.83 (±15.04)	<0.001	a	74.62 (±16.30)	0.03	a	72.84 (±14.95)	n.s.	a
	Cfb (%)		−0.75 (−2.08, 0.65)		a	−2.74 (−4.54, −1.08)		b	−0.33 (−2.95, 0.73)		a	−0.66 (−2.06, 0.14)		a
Waist circumferences (cm) (*n* ^†^ = 27, 27, 23, 27)		0	96.24 (±9.64)		a	95.69 (±12.98)		a	90.39 (±14.06)		a	90.80 (±12.36)		a
		12	93.80 (±9.34)	0.023	a	93.44 (±11.75)	0.019	a	90.43 (±12.33)	n.s.	a	89.48 (±12.85)	n.s.	a
	Cfb (%)		−2.38 (±5.23)		a	−2.12 (±4.85)		a	0.56 (±6.75)		a	−1.39 (±4.69)		a
Body fat (kg) (*n* ^†^ = 26, 27, 21, 27)		0	30.23 (±8.20)		a	33.50 (±7.07)		a	30.65 (±7.43)		a	28.49 (±7.95)		a
		12	29.23 (±7.78)	0.035	a	31.96 (±7.50)	<0.001	a	29.38 (±7.82)	0.018	a	27.50 (±8.55)	0.026	a
	Cfb (%)		−4.10 (−6.63, −0.49)		a	−5.62 (−8.51, −1.33)		a	−2.33 (−7.50, 0.52)		a	−4.32 (−7.16, 1.09)		a
Body water (l) (*n* ^†^ = 26, 27, 21, 27)		0	39.45 (±7.39) 38.50 (33.70, 44.70)		a	37.40 (33.65, 41)		a	34.10 (32.20, 42.10)		a	38.68 (±7.34) 39.10 (32.25, 45.40)		a
		12	39.43 (±7.09) 37.75 (34.20, 42.48)	n.s.	a	37.90 (33.75, 40.45)	n.s.	a	34.30 (32.40, 40.40)	n.s.	a	38.75 (±7.71) 37.50 (32.20, 43.20)	n.s.	a
	Cfb (%)		0.18 (±4.09)		a	−0.68 (±3.21)		a	0.46 (±2.94)		a	0.09 (±3.60)		a
Lean body mass (kg) (*n* ^†^ = 26, 27, 21, 27)		0	53.92 (±10.09) 52.60 (46.03, 61.10)		a	51.10 (45.95, 56.00)		a	46.50 (44.00, 57.50)		a	52.84 (±10.04) 53.40 (44.10, 62.00)		a
		12	53.87 (±9.69) 51.60 (46.73, 58.05)	n.s.	a	51.80 (46.10, 55.25)	n.s.	a	46.80 (44.30, 55.20)	n.s.	a	52.93 (±10.52) 51.20 (44.00, 59.05)	n.s.	a
	Cfb (%)		0.15 (±4.06)		a	−0.72 (±3.24)		a	0.46 (±2.89)		a	0.09 (±3.61)		a
BMI (kg/m^2^) (*n* ^†^ = 27, 27, 21, 28)		0	25.77 (24.44, 28.70)		a	27.90 (±4.57) 27.29 (24.45, 30.48)		a	25.55 (±4.54) 24.90 (23.00, 27.72)		a	23.90 (22.60, 28.23)		a
		12	25.70 (24.07, 28.80)	0.008	a	27.12 (±4.70) 26.12 (23.78, 29.39)	<0.001	a	25.22 (±4.42) 24.40 (22.30, 27.80)	0.030	a	23.95 (22.34, 28.73)	n.s.	a
	Cfb (%)		−1.45 (±2.70)		a	−2.86 (±2.75)		a	−1.24 (±2.47)		a	−0.91 (±3.15)		a
Systolic blood pressure (mmHG) (*n* ^†^ = 27, 27, 23, 28)		0	139.93 (±19.97) 137.00 (127.00, 152.00)		a	142.30 (±19.60)		a	138.48 (±21.73)		a	140.82 (±23.98)		a
		12	136.00 (122.50, 143.50)	0.043	a	136.96 (±18.85) 135.00 (125.50, 148.50)	n.s.	a	131.00 (±16.77) 129.00 (119.50, 142.50)	0.021	a	133.14 (±20.62) 130.00 (119.75, 146.00)	0.005	a
	Cfb (%)		−2.93 (±9.48)		a	−3.31 (±8.49)		a	−4.55 (±9.95)		a	−4.75 (±9.63)		a
Diastolic blood pressure (mmHG) (*n* ^†^ = 27, 27, 23, 28)		0	85.00 (78.00, 92.50)		a	88.00 (80.50, 93.00)		a	86.35 (±11.23) 84.00 (79.00, 95.50)		a	85.11 (±10.14) 85.00 (76.75, 95.25)		a
		12	84.00 (80.00, 89.00)	0.043	a	84.00 (77.50, 90.50)	n.s.	a	81.22 (±7.45) 81.00 (75.00, 87.50)	0.005	a	82.00 (±9.73) 80.00 (74.50, 88.25)	0.032	a
	Cfb (%)		−2.67 (±7.36)		a	−2.21 (±11.41)		a	−5.12 (±9.27)		a	−3.26 (±8.15)		a
Heart rate (bpm) (*n* ^†^ = 27, 27, 23, 28)		0	70.44 (±11.31) 70.00 (62.50, 79.00)		a	66.00 (61.00, 70.50)		a	67.26 (±10.98) 67.00 (60.00, 73.00)		a	66.00 (61.00, 69.25)		a
		12	69.67 (±9.36)	n.s.	a	68.07 (±10.13) 68.00 (62.00, 75.00)	n.s.	a	65.39 (±12.15)	n.s.	a	66.57 (±10.34) 65.50 (60.00, 71.50)	n.s.	a
	Cfb (%)		−0.06 (±11.88)		a	2.21 (±12.23)		a	−2.50 (±12.22)		a	−0.09 (±14.52)		a

* Variables expressed as mean (±SD) and/or as median (25th, 75th percentile) depending on the statistical tests that were performed; ◊ groups without a common letter are significantly different, *p* < 0.05; ∆ differences within groups comparing baseline and week 12; ^†^ number of data sets used to calculate shown data sorted by group from A to D. Abbreviations: BMI, body mass index; Cfb (%), percentage change from baseline; CRP, c-reactive protein; HbA1c, glycated hemoglobin A1c; HDL, high-density lipoprotein; LDL, low-density lipoprotein; MDA-LDL, malondialdehyde-modified-low-density lipoprotein.

## Data Availability

Requests to access the data sets should be directed to the corresponding author.

## Supplementary Materials

The following supporting information can be downloaded at: https://www.mdpi.com/article/10.3390/nu15204461/s1, Table S1: Biochemical methods; Table S2: Energy and nutrient intake of the study participants in the week before the start of the intervention period (self-reports, 7 days); Table S3: Comparison of erythrocyte fatty acids at baseline (week 0), after weeks 4 and 8 and at the end of the intervention period (week 12); Table S4: Comparison of percentage change from baseline without diet group subdivision but with split by sex differences, EPA baseline status and LA change throughout the study. Reference [78] is cited in the Supplementary Materials.
