# Supplementary material for: Evaluation of Influencing Factors on Metabolism of Land-Based n-3 Poly Unsaturated Fatty Acids—The KoALA Study
Source: Nutrients. 2023 Oct 20;15(20):4461. doi: 10.3390/nu15204461 (PMC10610546; doi:10.3390/nu15204461)
Supplement: Supplementary file 1 [file nutrients-15-04461-s001.zip › nutrients-2623317-supplementary.pdf]

Supplementary data

Table S1. Biochemical methods.

Parameter	Instrument	Reference range
	Plasma, serum	
Total cholesterol (mmol/l) *	Abbott Architect CI 16200 analyzer (Abbott, Wiesbaden, Germany)	<5.2
LDL cholesterol (mmol/l) *	Abbott Architect CI 16200 analyzer (Abbott, Wiesbaden, Germany)	<3.35
HDL cholesterol (mmol/l) *	Abbott Architect CI 16200 analyzer (Abbott, Wiesbaden, Germany)	>1.03
Triglycerides (mmol/l) *	Abbott Architect CI 16200 analyzer (Abbott, Wiesbaden, Germany)	<1.7
Apolipoprotein A (g/l) ◊	COBAS INTEGRA 400 plus System (Roche Diagnostics Ltd., Rotkreuz, Switzerland)	women: 1.08 – 2.25<br>men: 1.04 – 2.02
Apolipoprotein B (g/l) ◊	COBAS INTEGRA 400 plus System (Roche Diagnostics Ltd., Rotkreuz, Switzerland)	women: 0.60 – 1.17<br>men: 0.66 – 1.33
High-sensitivity CRP (mg/d) *	Abbott Architect CI 16200 analyzer (Abbott, Wiesbaden, Germany)	≤0.3
Homocysteine (µmol/l) *	HPLC (Shimadzu, Kyoto, Japan)	5 – 15
Vitamin E (µmol/l) *	HPLC (Shimadzu, Kyoto, Japan)	11.6 – 46.4
Glucose (mmol/l) *	Abbott Architect CI 16200 analyzer (Abbott, Wiesbaden, Germany)	18 – 60 y: 4.1 – 5.9<br>60 – 90 y: 4.6 – 6.4
Insulin (mU/l) *	Abbott Architect CI 16200 analyzer (Abbott, Wiesbaden, Germany)	3 – 25
HbA1c (%) *	Tosoh HLC-723G11 (Sysmex, Norderstedt, Germany)	4.5 – 6.1
Lipoprotein(a) (nmol/l) *	Abbott Architect CI 16200 analyzer (Abbott, Wiesbaden, Germany)	<72
MDA-LDL cholesterol (U/l) ◊	Manual MDA-LDL ELISA, ImmBioMed, 2019	No information available
Calcium (mmol/l) *	Abbott Architect CI 16200 analyzer (Abbott, Wiesbaden, Germany)	2.15 – 2.50
Potassium (mmol/l) *	Abbott Architect CI 16200 analyzer (Abbott, Wiesbaden, Germany)	3.4 – 4.5
Ferritin (µg/l) *	Abbott Architect CI 16200 analyzer (Abbott, Wiesbaden, Germany)	women: 13 – 150<br>men: 30 – 400
Transferrin (g/l) *	Abbott Architect CI 16200 analyzer (Abbott, Wiesbaden, Germany)	2.0 – 3.6
Folic acid (ng/ml) *	Abbott Architect CI 16200 analyzer (Abbott, Wiesbaden, Germany)	3.9 – 26.8 [78]
Biotin (ng/l) ◊	ELISA	> 250

Table S1. Continued.

Parameter	Instrument	Reference range
	Plasma, serum	
Holo-transcobalamin (pmol/l) *	Abbott Architect CI 16200 analyzer (Abbott, Wiesbaden, Germany)	> 37.52
Vitamin A (µmol/l) *	HPLC (Shimadzu, Kyoto, Japan)	1.46 – 2.84 [78]
Vitamin B_2 (µg/l) *	HPLC (Shimadzu, Kyoto, Japan)	180 – 295
Vitamin B_6 (nmol/l) *	HPLC (Shimadzu, Kyoto, Japan)	14.6 – 72.8
Vitamin B_{12} (pmol/l) *	Abbott Architect CI 16200 analyzer (Abbott, Wiesbaden, Germany)	197 – 7712
Vitamin C (mg/l) *	HPLC (Shimadzu, Kyoto, Japan)	4 – 15
Vitamin D (nmol/l) *	Abbott Architect CI 16200 analyzer (Abbott, Wiesbaden, Germany)	> 75
Vitamin E (µmol/l) *	HPLC (Shimadzu, Kyoto, Japan)	11.6 – 46.4
	Erythrocytes	
Fatty acids (% FAME) $	GC-17V3 (Shimadzu, Duisburg, Germany)	No information available

* Measured by Institute of Clinical Chemistry and Laboratory Diagnostics, University Hospital Jena, Jena, Germany; ◊ measured by Dianovis GmbH, Greiz, Germany; $ measured by Institute of Nutritional Sciences, Friedrich Schiller University, Jena, Germany. Abbreviations: CRP, c-reactive protein; FAME, fatty acid methyl ester; HDL, high-density lipoprotein; HbA1c, glycated hemoglobin A1c; LDL, low-density lipoprotein; MDA-LDL, malondialdehyd-modified-low-density lipoprotein

Table S2. Energy and nutrient intake of the study participants in the week before the start of the intervention period (self-reports, 7 days).

	**High linoleic acid** (n = 25)		**Low linoleic acid** (n = 25)		**High milk** (n = 21)		**Control** (n = 26)	
**Energy and nutrients**	**Characteristics ***	**◊**	**Characteristics ***	**◊**	**Characteristics ***	**◊**	**Characteristics ***	**◊**
**Energy** (kcal/day)	2065 (1846, 2600)	a	2219 (1724, 2535)	a	2098 (1846, 2624)	a	2204 (1888, 2532)	a
**Carbohydrate** (g/day)	224 (192, 294)	a	213 (182, 247)	a	220 (189, 273)	a	238 (175, 273)	a
**Fiber** (g/day)	23.9 (19.5, 27.1)	a	28.4 (20.7, 34.1)	a	24.7 (19.9, 40.1)	a	27.0 (20.0, 32.9)	a
**Total sugar** (g/day)	108 (87, 155)	a	93 (77, 133)	a	108 (83, 137)	a	109 (83, 146)	a
**Sucrose** (g/day)	50.7 (39.0, 73.6)	a	43.5 (31.5, 57.2)	a	52.6 (37.2, 62.0)	a	52.8 (43.9, 62.8)	a
**Glucose** (g/day)	19.4 (14.3, 25.6)	a	18.8 (14.2, 23.6)	a	22.3 (14.4, 24.9)	a	18.1 (15.1, 26.8)	a
**Fructose** (g/day)	24.0 (18.4, 32.3)	a	23.4 (19.6, 29.2)	a	26.1 (18.7, 35.0)	a	24.0 (20.1, 32.2)	a
**Alcohol** (g/day)	7.4 (2.4, 14.0)	a	7.4 (3.8, 15.2)	a	6.0 (0.2, 15.1)	a	4.1 (2.2, 9.9)	a
**Protein** (g/day)	82 (67, 109)	a	87 (69, 106)	a	87 (78, 102)	a	91 (81, 104)	a
**Fat** (g/day)	83 (63, 100)	a	87 (73, 118)	a	88 (70, 101)	a	92 (74, 106)	a
**SFA** (g/day)	32.2 (28.9, 42.3)	a	37.6 (30.7, 44.6)	a	33.9 (28.6, 41.8)	a	36.1 (29.6, 41.3)	a
**MUFA** (g/day)	28.5 (22.2, 36.8)	a	31.5 (25.7, 45.6)	a	32.3 (25.2, 40.2)	a	32.5 (27.3, 38.3)	a
**PUFA** (g/day)	11.4 (9.3, 16.6)	a	12.8 (9.2, 16.4)	a	12.4 (9.9, 22.2)	a	12.5 (10.2, 16.8)	a
**C-18:2$_{n6}$** (g/day)	8.8 (7.6, 13)	a	10.4 (7.4, 14.5)	a	10.0 (7.7, 15.4)	a	10.2 (7.6, 12.8)	a
**C-18:3$_{n3}$** (g/day)	1.2 (1.0, 1.6)	a	1.4 (1.1, 1.5)	a	1.3 (0.9, 1.6)	a	1.3 (1.1, 1.8)	a
**C-20:5$_{n3}$** (g/day)	0.2 (0.0, 0.5)	a	0.2 (0.1, 0.3)	a	0.2 (0.1, 0.4)	a	0.3 (0.1, 0.5)	a
**C-22:6n$_3$** (g/day)	0.2 (0.1, 0.3)	a	0.2 (0.1, 0.4)	a	0.3 (0.1, 0.5)	a	0.4 (0.2, 0.5)	a
**C-20:4$_{n6}$** (g/day)	0.2 (0.1, 0.3)	a	0.2 (0.1, 0.3)	a	0.2 (0.1, 0.3)	a	0.2 (0.1, 0.3)	a
**Vitamin A** (mg/day)	0.5 (0.4, 1.0)	a	0.6 (0.4, 1.2)	a	0.4 (0.3, 0.5)	a	0.5 (0.4, 0.7)	a
**Vitamin B$_1$** (mg/day)	1.5 (1.2, 2.0)	a	1.6 (1.3, 2.2)	a	1.9 (1.2, 2.1)	a	1.5 (1.3, 1.9)	a
**Vitamin B$_2$** (mg/day)	1.6 (1.3, 1.8)	a	1.6 (1.2, 1.9)	a	1.5 (1.4, 1.8)	a	1.7 (1.4, 2.0)	a
**Vitamin B$_6$** (mg/day)	1.9 (1.5, 2.5)	a	2.0 (1.7, 2.2)	a	2.1 (1.8, 2.4)	a	2.0 (1.7, 2.5)	a
**Vitamin B$_{12}$** (μg/day)	5.6 (4.8, 7.9)	a	5.4 (4.4, 6.6)	a	5.2 (4.1, 6.5)	a	5.7 (4.4, 7.4)	a
**Vitamin C** (mg/day)	124 (94, 145)	a	122 (88, 176)	a	139 (108, 161)	a	130 (92, 149)	a
**Vitamin D** (μg/day)	3.7 (2.2, 6.9)	a	2.0 (1.2, 4.4)	a	3.2 (2.3, 4.4)	a	3.9 (2.0, 6.7)	a
**Vitamin E** (mg/day)	9.1 (7.8, 13.7)	a	10.7 (9.0, 15.8)	a	10.5 (9.2, 17.2)	a	11.9 (9.4, 14.4)	a
**Vitamin K** (mg/day)	0.1 (0.1, 0.2)	a	0.2 (0.1, 0.2)	a	0.1 (0.1, 0.2)	a	0.2 (0.1, 0.2)	a
**Calcium** (mg/day)	832 (654, 932)	a	788 (645, 980)	a	811 (647, 1002)	a	844 (671, 1145)	a
**Magnesium** (mg/day)	346 (290, 437)	a	391 (320, 457)	a	353 (292, 478)	a	382 (332, 458)	a
**Potassium** (mg/day)	3389 (2800, 4096)	a	3601 (2858, 4036)	a	3512 (3011, 4243)	a	3605 (3325, 4431)	a
**Iron** (mg/day)	12.7 (10.0, 15.8)	a	12.4 (10.7, 14.5)	a	12.0 (9.5, 15.3)	a	12.8 (11.1, 14.7)	a
**Zinc** (mg/day)	10.4 (8.4, 13.9)	a	11.4 (8.9, 13.8)	a	11.2 (9.5, 15.1)	a	11.7 (11.3, 14.9)	a
**Sodium** (mg/day)	2310 (1820, 3195)	a	2180 (1810, 2700)	a	2131 (1741, 2704)	a	2061 (1694, 2801)	a
**Chloride** (mg/day)	4073 (2930, 4675)	a	3620 (2815, 4069)	a	3578 (2943, 4335)	a	3444 (2826, 4860)	a
**Phosphor** (mg/day)	1410 (1080, 1623)	a	1424 (1075, 1652)	a	1354 (1120, 1750)	a	1528 (1252, 1794)	a
**Iodine** (μg/day)	91 (76, 137)	a	106 (72, 130)	a	99 (73, 116)	a	106 (71, 134)	a
**Copper** (mg/day)	1.8 (1.6, 2.3)	a	2.0 (1.6, 2.3)	a	1.7 (1.5, 2.6)	a	2.0 (1.7, 2.5)	a
**Manganese** (mg/day)	4.5 (3.6, 5.8)	a	4.7 (3.8, 6.0)	a	4.1 (3.3, 7.6)	a	4.9 (3.9, 7.2)	a

* Variables expressed as mean (±SD) and/or as median (25th, 75th percentile) depending on the statistical test that was performed; ◊ groups without a common letter are significantly different, $p < 0.05$. Abbreviations: MUFA, monounsaturated fatty acids; PUFA, polyunsaturated fatty acids; SFA, saturated fatty acids

Table S3. Comparison of erythrocyte fatty acids at baseline (week 0), after weeks 4 and 8 and at the end of the intervention period (week 12).

**Erythrocyte fatty acids**<br>(% FAME)	**Week**	**High linoleic acid**<br>(*n* = 27)			**Low linoleic acid**<br>(*n* = 27)			**High milk**<br>(*n* = 22)			**Control**<br>(*n* = 27)		
		**Characteristics ***	**Δ**	**◊**	**Characteristics ***	**Δ**	**◊**	**Characteristics ***	**Δ**	**◊**	**Characteristics ***	**Δ**	**◊**
C-14:0	0	0.16 (±0.07)	a	a	0.17 (±0.06)	a	a,b	0.20 (±0.06)	a	b	0.25 (±0.07)<br>0.25 (0.19, 0.30)	a	c
	4	0.19 (±0.05)	a	a	0.19 (±0.06)	a,b	a	0.21 (±0.06)	a	a	0.25 (±0.07)<br>0.25 (0.21, 0.31)	a	b
	8	0.20 (±0.04)<br>0.21 (0.18, 0.22)	a	a	0.20 (±0.05)<br>0.19 (0.17, 0.24)	b	a	0.24 (±0.09)<br>0.23 (0.19, 0.26)	a	a	0.27 (0.23, 0.33)	b	b
	12	0.20 (±0.03)	a	a	0.19 (±0.03)	a,b	a	0.22 (±0.08)	a	a	0.29 (±0.07)<br>0.28 (0.25, 0.34)	b	b
Cfb (%)		33.03 (0.40, 81.76)		a	14.33 (−12.35, 61.48)		a	−7.24 (−20.07, 66.48)		a	21.95 (8.61, 28.45)		a
C-15:0	0	0.12 (±0.03)<br>0.11 (0.09, 0.14)	a	a	0.11 (0.10, 0.13)	a	a	0.13 (0.12, 0.19)	a	b	0.16 (±0.03)<br>0.16 (0.14, 0.18)	a	b
	4	0.14 (±0.02)	b,c	a	0.13 (±0.03)<br>0.13 (0.12, 0.15)	b,c	a	0.15 (±0.03)<br>0.14 (0.12, 0.18)	a	a	0.17 (±0.03)	a	b
	8	0.14 (±0.02)<br>0.14 (0.12, 0.15)	b	a,b	0.14 (0.13, 0.15)	b	a	0.16 (0.13, 0.19)	a	b,c	0.18 (±0.04)<br>0.18 (0.16, 0.20)	b	c
	12	0.13 (±0.02)	a	a	0.12 (±0.02)<br>0.12 (0.11, 0.13)	a,c	a	0.15 (±0.05)<br>0.15 (0.11, 0.17)	a	b	0.19 (±0.04)	b	c
Cfb (%)		10.71 (−8.08, 35.74)		a	10.73 (−7.42, 27.21)		a	−13.50 (−19.21, 31.98)		a	15.82 (8.17, 29.73)		a
C-16:0	0	16.58 (±2.71)	a	a	16.78 (±2.70)	a	a	21.98 (±2.84)<br>21.23 (20.07, 23.75)	a	b	21.13 (±1.74)<br>20.81 (19.92, 21.98)	a	b
	4	18.65 (±2.24)<br>18.7 (17.55, 20.23)	b	a	18.96 (±2.28)<br>19.09 (18.05, 20.54)	b	a	21.09 (19.13, 23.56)	a	b	22.57 (20.60, 22.88)	b	b
	8	20.17 (±0.88)	c	a	20.35 (±1.08)	c	a	23.10 (±3.96)<br>23.68 (20.86, 24.91)	a	b	23.04 (±1.37)<br>23.14 (22.13, 23.80)	b,c	b
	12	20.25 (±1.11)<br>20.02 (19.49, 21.12)	c	a	20.11 (±0.77)<br>20.12 (19.63, 20.49)	c	a	23.37 (19.37, 25.60)	a	b	23.66 (22.57, 24.68)	c	c
Cfb (%)		23.33 (7.84, 42.58)		a	22.29 (4.36, 38.32)		a	−1.45 (−8.83, 22.04)		b	11.93 (8.63, 19.38)		a,b

Table S3. Continued

Erythrocyte fatty acids (% FAME)	Week	High linoleic acid (*n* = 27)			Low linoleic acid (*n* = 27)			High milk (*n* = 22)			Control (*n* = 27)		
		**Characteristics ***	**Δ**	**◊**	**Characteristics ***	**Δ**	**◊**	**Characteristics ***	**Δ**	**◊**	**Characteristics ***	**Δ**	**◊**
C-17:0	0	0.25 (±0.03)	a	a	0.25 (±0.03)<br>0.25 (0.23, 0.27)	a	a	0.28 (±0.05)<br>0.27 (0.24, 0.31)	a	b	0.29 (±0.03)	a	b
	4	0.27 (±0.03)<br>0.27 (0.24, 0.29)	b	a	0.26 (0.25, 0.28)	b,c	a	0.28 (0.25, 0.30)	a	a,b	0.30 (±0.03)<br>0.30 (0.27, 0.33)	a	b
	8	0.27 (±0.04)<br>0.27 (0.25, 0.30)	b	a	0.27 (0.25, 0.29)	b	a	0.31 (0.29, 0.34)	b	b	0.31 (±0.04)<br>0.31 (0.29, 0.33)	b	b
	12	0.25 (±0.03)<br>0.25 (0.24, 0.28)	a	a	0.25 (0.24, 0.28)	a,c	a	0.30 (0.28, 0.33)	b	b	0.32 (±0.04)<br>0.32 (0.29, 0.35)	b	b
	Cfb (%)	1.32 (−3.52, 9.32)		a	3.78 (−1.11, 8.57)		a	8.14 (3.08, 20.65)		a	10.49 (1.55, 17.90)		a
C-18:0	0	14.42 (±1.26)	a	a	14.50 (±1.57)	a	a	12.38 (±1.99)<br>11.95 (11.10, 13.56)	a	b	14.08 (±1.85)	a	a
	4	12.16 (±1.43)	b	a	12.43 (±1.46)	b	a	11.42 (±1.24)<br>11.12 (10.61, 12.01)	a	a	11.89 (±1.21)	b	a
	8	11.29 (±0.70)<br>11.34 (10.81, 11.73)	c	a	11.44 (±0.79)<br>11.45 (10.93, 11.93)	c	a	14.12 (12.66, 14.87)	b	b	12.45 (±1.40)<br>12.53 (11.56, 13.52)	b	c
	12	11.90 (±1.00)<br>12.28 (11.44, 12.59)	b	a	11.70 (±0.94)<br>11.56 (11.08, 12.29)	c	a	12.06 (11.16, 12.99)	a	a	11.71 (±1.36)<br>11.45 (10.69, 12.78)	b	a
	Cfb (%)	−16.56 (−26.00, −10.62)		a	−20.62 (−26.92, −14.52)		a	−3.86 (−13.90, 8.69)		b	−14.13 (−24.25, −6.09)		a
C-18:1c9	0	13.95 (±1.03)<br>14.19 (13.24, 14.52)	a	a	14.08 (±1.18)	a	a	16.41 (±1.24)<br>16.27 (15.56, 17.49)	a	b	15.30 (±1.29)	a	c
	4	14.57 (±0.85)<br>14.67 (14.01, 15.20)	b	a	14.86 (±1.05)	b	a,b	15.59 (±1.20)	b	b	15.25 (±1.30)	a	a,b
	8	15.13 (±0.73)<br>15.15 (14.61, 15.55)	b,c	a	15.62 (±1.02)	c	a	15.84 (±1.19)<br>15.94 (15.11, 16.87)	a,b	a	15.34 (±1.38)	a	a
	12	14.94 (14.71, 16.03)	c	a	15.90 (±1.05)<br>16.14 (14.87, 16.65)	d	a,b	16.62 (15.55, 17.18)	a	b	15.56 (±1.02)<br>15.48 (15, 16.31)	a	a,b
	Cfb (%)	8.54 (4.74, 16.41)		a	10.46 (9.03, 16.77)		a	0.88 (−5.25, 3.85)		b	2.38 (−2.95, 8.35)		b
C-18:2c9c12 (LA)	0	10.02 (±1.16)<br>10.09 (9.47, 10.62)	a	a	10.31 (±1.05)<br>10.31 (9.63, 10.72)	a	a	11.85 (10.97, 12.48)	a	b	11.02 (10.46, 11.52)	a	b
	4	11.76 (±1.36)	b	a	11.19 (±1.22)	b	a	11.94 (±1.49)<br>11.77 (11.28, 13.04)	a	a	11.78 (±1.42)<br>11.73 (11.01, 12.39)	b	a
	8	12.83 (±1.42)<br>12.99 (11.92, 13.83)	c	a	12.20 (±0.99)<br>12.42 (11.56, 12.89)	c	a,b	12.10 (11.09, 12.63)	a	b	11.78 (11.08, 12.46)	b	b
	12	12.71 (±1.45)	c	a	12.35 (±0.87)	c	a	11.97 (±1.69)<br>12.13 (11.55, 12.68)	a	a	11.87 (±1.24)<br>11.85 (11.10, 12.42)	b	a
	Cfb (%)	27.30 (22.09, 33.03)		a	21.74 (13.50, 28.30)		a	5.02 (−0.99, 7.88)		b	7.47 (3.22, 12.59)		b

Table S3. Continued.

Erythrocyte fatty acids (% FAME)		Week	High linoleic acid (n = 27)			Low linoleic acid (n = 27)			High milk (n = 22)			Control (n = 27)		
			**Characteristics ***	**Δ**	**◊**	**Characteristics ***	**Δ**	**◊**	**Characteristics ***	**Δ**	**◊**	**Characteristics ***	**Δ**	**◊**
aC-18:3c9c12c15 (ALA)<br>(n [†] (Control) = 26)		0	0.14 (0.12, 0.17)	a	a	0.17 (0.14, 0.23)	a	a	0.14 (0.12, 0.17)	a	a	0.17 (0.13, 0.23)	a	a
		4	0.55 (±0.19)<br>0.56 (0.40, 0.70)	b	a	0.60 (±0.27)<br>0.54 (0.43, 0.82)	b	a	0.61 (±0.24)<br>0.61 (0.41, 0.77)	b	a	0.58 (±0.18)<br>0.57 (0.47, 0.71)	b	a
		8	0.67 (±0.27)<br>0.69 (0.52, 0.74)	c	a	0.80 (±0.39)<br>0.77 (0.52, 1.06)	c	a	0.65 (±0.32)<br>0.64 (0.39, 0.81)	b	a	0.64 (±0.22)<br>0.60 (0.48, 0.79)	b	a
		12	0.79 (±0.31)<br>0.78 (0.59, 1.02)	d	a	0.81 (±0.39)<br>0.82 (0.55, 1.12)	c	a	0.69 (±0.28)<br>0.75 (0.50, 0.92)	b	a	0.70 (±0.23)<br>0.72 (0.57, 0.79)	b	a
	Cfb (%)		465.08 (±286.92)		a	354.34 (±223.79)		a	378.33 (±250.97)		a	296.30 (±158.53)		a
C-20:2c11c14		0	0.20 (0.19, 0.25)	a	a	0.23 (0.20, 0.25)	a	a	0.24 (±0.03)<br>0.23 (0.22, 0.26)	a	a	0.22 (0.18, 0.24)	a	a
		4	0.21 (0.18, 0.21)	a	a,c	0.19 (0.18, 0.21)	b	a	0.23 (±0.03)<br>0.22 (0.21, 0.24)	a	b	0.21 (0.20, 0.23)	a	b,c
		8	0.19 (0.18, 0.21)	a	a	0.19 (0.18, 0.21)	b	a	0.23 (±0.04)<br>0.23 (0.20, 0.25)	a	b	0.21 (0.19, 0.23)	a	a,b
		12	0.19 (0.17, 0.21)	a	a	0.19 (0.18, 0.21)	b	a	0.24 (±0.05)<br>0.23 (0.21, 0.26)	a	b	0.22 (0.19, 0.24)	a	b
	Cfb (%)		−7.58 (−18.78, −1.51)		a	−8.94 (−21.81, 1.39)		a,c	−1.72 (−12.00, 16.26)		b,c	−1.65 (−7.88, 7.10)		b
C-20:3c8c11c14		0	1.44 (1.38, 1.60)	a	a	1.54 (1.25, 1.65)	a	a	1.41 (1.26, 1.59)	a	a	1.39 (1.20, 1.59)	a	a
		4	1.19 (1.07, 1.37)	a	a	1.15 (0.99, 1.41)	b	a	1.20 (1.11, 1.35)	b	a	1.17 (1.02, 1.36)	b	a
		8	1.07 (1.01, 1.26)	b	a	1.14 (0.97, 1.30)	b	a	1.21 (1.07, 1.38)	b	a	1.15 (1.01, 1.32)	b	a
		12	1.10 (1.00, 1.24)	b	a	1.10 (0.98, 1.25)	b	a	1.32 (1.19, 1.47)	a,b	b	1.07 (0.99, 1.32)	b	a
	Cfb (%)		−21.86 (−34.38, −15.79)		a	−15.70 (−33.06, −7.93)		a	−7.14 (−20.83, 4.24)		b	−16.65 (−23.01, −13.99)		a,b
C-20:4c5c8c11c14 (ARA)		0	15.73 (±1.73)<br>15.50 (14.52, 16.72)	a	a	15.36 (13.96, 16.22)	a	a	14.78 (13.82, 15.62)	a	a	14.30 (13.82, 15.47)	a	a
		4	14.25 (±1.53)<br>13.86 (13.25, 15.10)	b	a	14.10 (13.27, 14.77)	b	a	15.00 (13.56, 16.21)	a	a	13.19 (12.70, 13.69)	b	b
		8	13.46 (±1.24)<br>13.64 (12.66, 14.13)	c	a,b	13.11 (12.44, 13.67)	c	a,b	14.20 (13.06, 15.92)	a	a	12.92 (12.26, 13.58)	b	b
		12	13.39 (±1.17)<br>13.62 (12.41, 14.05)	c	a	13.35 (12.65, 14.09)	b,c	a	13.96 (12.64, 15.92)	a	a	12.40 (11.48, 13.29)	c	b
	Cfb (%)		−15.55 (−20.20, −9.14)		a	−10.42 (−17.93, −6.52)		a,b	−4.80 (−12.93, 4.97)		b	−13.52 (−18.61, −7.94)		a
C-20:4n3 (ETA)<br>(n [†] (Control) = 22)		0	0.09 (0.07, 0.12)	a	a	0.09 (0.07, 0.14)	a	a	0.07 (0.05, 0.08)	a	b	0.08 (0.06, 0.11)	a	a,b
		4	0.12 (0.10, 0.15)	a,b	a	0.14 (0.09, 0.17)	a,b	a	0.12 (0.10, 0.18)	b	a	0.13 (0.09, 0.16)	b	a
		8	0.12 (0.10, 0.15)	b	a	0.14 (0.11, 0.17)	b	a	0.13 (0.09, 0.16)	b	a	0.13 (0.11, 0.16)	b	a
		12	0.16 (0.11, 0.18)	b	a	0.15 (0.13, 0.19)	b	a	0.16 (0.10, 0.20)	b	a	0.14 (0.12, 0.18)	b	a
	Cfb (%)		72.75 (14.74, 157.73)		a,b	53.87 (18.57, 146.26)		a	140.21 (93.21, 210.24)		b	83.58 (53.3, 119.51)		a,b

Table S3. Continued.

Erythrocyte fatty acids (% FAME)	Week	High linoleic acid (n = 27) Characteristics *	Δ	◊	Low linoleic acid (n = 27) Characteristics *	Δ	◊	High milk (n = 22) Characteristics *	Δ	◊	Control (n = 27) Characteristics *	Δ	◊
C-20:5n3 (EPA)	0	0.96 (±0.34)<br>0.90 (0.74, 1.21)	a	a	0.91 (0.79, 1.26)	a	a	0.84 (±0.31)<br>0.86 (0.62, 1.03)	a	a	0.91 (0.75, 1.37)	a	a
	4	1.20 (±0.25)<br>1.19 (1.06, 1.39)	b	a	1.16 (0.95, 1.36)	b	a	1.16 (±0.38)<br>1.13 (1.01, 1.52)	b	a	1.32 (0.87, 1.41)	b	a
	8	1.31 (±0.35)<br>1.25 (1.09, 1.46)	c	a	1.37 (1.06, 1.61)	c	a	1.11 (±0.30)<br>1.11 (0.95, 1.37)	b	a	1.24 (0.99, 1.51)	b	a
	12	1.51 (±0.46)	d	a	1.51 (±0.41)<br>1.55 (1.34, 1.80)	c	a	1.36 (±0.52)	c	a	1.46 (±0.51)<br>1.42 (1.21, 1.63)	b	a
	Cfb (%)	68.84 (28.20, 85.26)		a	65.94 (16.93, 81.34)		a	72.84 (34.36, 107.80)		a	37.17 (15.32, 68.61)		a
C-22:4n6	0	3.25 (±0.77)<br>3.08 (2.66, 3.68)	a	a	3.23 (±0.86)<br>3.12 (2.55, 3.88)	a	a	2.97 (±0.64)<br>3.05 (2.59, 3.43)	a	a	2.45 (±0.48)	a	b
	4	2.71 (2.22, 2.90)	b	a	2.56 (2.29, 3.13)	a,b	a	2.87 (2.5, 3.62)	a	a	2.32 (±0.45)<br>2.27 (2.01, 2.61)	b	b
	8	2.34 (2.14, 2.72)	c	a,b	2.54 (2.22, 2.73)	b	a,b	2.62 (2.25, 3.17)	a	a	2.25 (±0.46)<br>2.30 (1.95, 2.51)	b	b
	12	2.28 (±0.40)<br>2.30 (1.94, 2.48)	c	a	2.42 (±0.38)<br>2.47 (2.11, 2.68)	b	a	2.87 (±0.85)<br>2.73 (2.46, 3.26)	a	b	2.14 (±0.48)	c	a
	Cfb (%)	−30.21 (−38.99, −14.09)		a	−22.85 (−35.85, −11.03)		a,c	−1.97 (−18.69, 12.07)		b	−12.25 (−17.56, −1.50)		b,c
C-22:5n6	0	0.49 (0.41, 0.62)	a	a	0.43 (0.32, 0.61)	a	a,b	0.36 (0.32, 0.42)	a	b	0.44 (0.35, 0.53)	a	a,b
	4	0.33 (0.30, 0.37)	b	a	0.31 (0.25, 0.36)	b	a	0.35 (0.29, 0.38)	a	a	0.33 (0.28, 0.41)	b	a
	8	0.29 (0.26, 0.34)	c	a,b	0.20 (0.22, 0.30)	c	b	0.31 (0.27, 0.41)	a	a	0.34 (0.28, 0.39)	b	a
	12	0.27 (±0.04)<br>0.27 (0.24, 0.29)	c	a,b	0.26 (±0.06)<br>0.27 (0.20, 0.29)	c	b	0.32 (±0.11)<br>0.29 (0.25, 0.35)	a	a	0.31 (±0.08)<br>0.31 (0.26, 0.36)	b	a
	Cfb (%)	−46.72 (−56.48, −36.62)		a	−39.13 (−49.77, −31.25)		a,c	−14.37 (−26.67, 0.72)		b	−31.98 (−46.20, −15.85)		c
C-22:5n3 (DPA)	0	3.00 (±0.69)<br>3.20 (2.51, 3.44)	a	a	2.97 (2.29, 3.44)	a,b	a	2.43 (2.01, 2.81)	a	b	2.36 (2.22, 2.72)	a	b
	4	2.69 (±0.57)<br>2.53 (2.42, 2.98)	a,b	a	2.63 (2.18, 2.91)	a	a	2.76 (2.39, 3.09)	a,b	a	2.46 (2.27, 2.60)	a	a
	8	2.61 (±0.36)	b	a	2.61 (±0.32)<br>2.57 (2.39, 2.91)	a	a	2.72 (±0.75)<br>2.56 (2.21, 3.20)	a	a	2.49 (±0.33)<br>2.51 (2.29, 2.64)	a,b	a
	12	2.81 (±0.39)<br>2.80 (2.66, 3.04)	a	a,b	2.88 (2.62, 3.04)	b	a,b	3.00 (2.77, 3.38)	b	a	2.65 (2.53, 2.83)	b	b
	Cfb (%)	−2.30 (±22.98)		a	1.81 (±23.42)		a	35.82 (±43.72)		b	11.27 (±14.86)		a

Table S3. Continued.

Erythrocyte fatty acids (% FAME)	Week	High linoleic acid (n = 27) Characteristics *	Δ	◊	Low linoleic acid (n = 27) Characteristics *	Δ	◊	High milk (n = 22) Characteristics *	Δ	◊	Control (n = 27) Characteristics *	Δ	◊
C-22:6n3 (DHA)	0	5.75 (±1.41)<br>5.73 (4.66, 6.54)	a	a	5.20 (4.40, 5.98)	a	a,c	4.18 (3.06, 4.95)	a	b	4.85 (±0.86)<br>4.76 (4.27, 5.47)	a	b,c
	4	4.53 (±1.01)	b	a	4.44 (±1.13)<br>4.32 (3.67, 4.96)	b	a	4.39 (±1.18)<br>4.18 (3.65, 5.33)	a	a	4.05 (±0.94)	b	a
	8	3.93 (±0.69)<br>4.06 (3.46, 4.23)	c	a	3.72 (3.08, 4.40)	c	a	3.53 (3.19, 4.35)	a	a	4.04 (±0.92)<br>4.04 (3.35, 4.61)	b	a
	12	3.87 (±0.66)	c	a	3.67 (±0.70)<br>3.67 (3.15, 4.21)	c	a	3.79 (±1.18)<br>3.91 (3.17, 4.67)	a	a	3.91 (±0.79)	b	a
Cfb (%)		−33.96 (−41.68, −20.05)		a	−35.47 (−39.61, −17.42)		a	−5.97 (−19.99, 14.69)		b	−18.66 (−29.33, −11.47)		b
SFA	0	33.63 (31.96, 34.58)	a	a	33.89 (31.87, 36.02)	a	a	36.28 (34.31, 38.94)	a	b	37.20 (36.45, 37.65)	a,b	b
	4	32.76 (31.78, 33.94)	a	a	34.00 (32.25, 34.89)	a	a	36.15 (33.74, 37.77)	a	b	36.37 (35.21, 37.24)	a	b
	8	33.88 (33.29, 34.43)	a	a	34.15 (33.44, 34.59)	a	a	39.30 (36.30, 40.91)	a	b	38.09 (37.24, 39.28)	b	b
	12	33.87 (33.07, 35.32)	a	a	33.77 (32.70, 34.67)	a	a	37.36 (33.48, 39.26)	a	b	37.78 (37.16, 38.63)	b	b
Cfb (%)		2.81 (−5.27, 9.70)		a	−0.38 (−7.79, 6.97)		a	0.00 (−11.49, 8.98)		a	1.77 (−0.82, 5.66)		a
MUFA	0	15.77 (±1.00)	a	a	15.90 (±1.21)	a	a	18.13 (±1.35)<br>18.11 (17.18, 19.34)	a	b	17.02 (±1.32)	a	c
	4	16.26 (±0.92)	b	a	16.61 (±1.15)	b	a	17.02 (±1.32)<br>17.07 (16.34, 17.83)	b	a	16.96 (±1.36)	a	a
	8	16.89 (±0.79)	c	a	17.42 (±1.25)	c	a	17.14 (±1.23)<br>17.14 (16.31, 18.15)	b	a	17.09 (±1.47)	a	a
	12	16.87 (±0.80)<br>16.60 (16.28, 17.49)	c	a	17.55 (±1.14)<br>17.77 (16.61, 18.33)	c	a,b	18.26 (17.41, 19.17)	a	b	17.40 (±1.06)<br>17.37 (16.65, 18.23)	a	a,b
Cfb (%)		7.30 (±7.55)		a	10.55 (±5.25)		a	−0.02 (±7.75)		b	2.57 (±7.14)		b
C-18:1c9/C-18:0	0	0.96 (0.92, 1.03)	a	a	1.01 (0.88, 1.07)	a	a	1.34 (1.25, 1.43)	a	b	1.11 (0.99, 1.24)	a	c
	4	1.22 (±0.17)<br>1.22 (1.15, 1.35)	b	a	1.21 (±0.18)<br>1.20 (1.07, 1.35)	b	a	1.38 (±0.18)<br>1.40 (1.28, 1.50)	a	b	1.29 (±0.14)<br>1.27 (1.19, 1.39)	b	a,b
	8	1.35 (1.31, 1.39)	b	a	1.36 (1.27, 1.46)	c	a	1.14 (1.07, 1.24)	b	b	1.24 (1.13, 1.33)	a,b	b
	12	1.26 (1.18, 1.41)	b	a	1.38 (1.24, 1.47)	b,c	a	1.34 (1.25, 1.49)	a	a	1.37 (1.20, 1.48)	b	a
Cfb (%)		35.69 (±25.96)		a,c	41.36 (±20.92)		a	1.66 (±21.63)		b	25.07 (±27.69)		c

Table S3. Continued.

Erythrocyte fatty acids (% FAME)	Week	High linoleic acid (n = 27)			Low linoleic acid (n = 27)			High milk (n = 22)			Control (n = 27)		
		**Characteristics ***	**Δ**	**◊**	**Characteristics ***	**Δ**	**◊**	**Characteristics ***	**Δ**	**◊**	**Characteristics ***	**Δ**	**◊**
PUFA	0	41.34 (±3.23)<br>41.02 (38.74, 43.55)	a	a	40.90 (±3.55)	a	a	39.55 (±3.80)<br>39.28 (36.79, 41.83)	a	a,b	38.65 (±2.00)<br>38.43 (37.49, 39.97)	a	b
	4	39.57 (38.06, 41.29)	a	a	39.02 (±2.63)<br>38.72 (37.26, 40.71)	b	a,b	40.93 (38.01, 42.68)	a	a	37.77 (36.59, 38.56)	b	b
	8	39.14 (±1.44)<br>39.37 (38.14, 40.27)	a	a	38.49 (±1.41)	b	a,b	39.27 (±4.47)<br>38.54 (37.50, 41.78)	a	a	37.39 (±1.60)<br>37.13 (36.55, 38.09)	b,c	b
	12	39.45 (38.59, 40.23)	a	a	38.93 (±1.04)<br>38.75 (38.17, 39.70)	b	a	40.32 (36.48, 42.82)	a	a	36.91 (35.95, 37.29)	c	b
Cfb (%)		−5.77 (−10.18, 3.04)		a	−4.00 (−9.84, 0.89)		a,b	2.80 (−4.24, 12.37)		b	−4.72 (−8.23, −1.93)		a
n-6 PUFA	0	31.30 (±1.82)	a	a,b	31.06 (±2.23)	a	a,b	32.01 (±3.27)<br>31.61 (29.97, 34.06)	a	a	30.03 (±1.80)<br>30.44 (29.01, 31.24)	a	b
	4	30.63 (±1.55)<br>30.47 (29.84, 31.22)	a,b	a,b	29.89 (±1.69)<br>29.97 (28.39, 31.41)	b	a,c	31.50 (29.91, 33.21)	a	b	29.23 (28.11, 30.15)	a,c	c
	8	30.38 (±1.26)	a,b	a	29.68 (±1.33)	b	a,b	30.74 (±3.14)<br>30.33 (29.44, 32.85)	a	a	28.76 (±1.83)<br>28.73 (27.45, 29.69)	b,c	b
	12	30.03 (±1.25)<br>30.51 (29.18, 30.89)	b	a	29.84 (±1.27)<br>29.47 (28.75, 30.86)	b	a	30.83 (29.18, 33.44)	a	a	28.06 (26.89, 29.05)	b	b
Cfb (%)		−4.06 (−7.91, 0.17)		a,b	−3.57 (−6.14, −0.77)		a,b	−0.23 (−8.36, 5.23)		a	−7.18 (−9.00, −4.71)		b
n-3 PUFA	0	9.57 (8.59, 11.45)	a	a	9.47 (7.86, 10.50)	a	a,c	7.54 (±1.87)<br>7.23 (5.95, 9.10)	a	b	8.61 (±1.38)<br>8.60 (7.49, 9.49)	a	b,c
	4	9.12 (±1.49)<br>8.83 (8.21, 9.70)	a	a	9.03 (±1.85)<br>8.84 (7.83, 9.60)	a	a	9.20 (±1.62)	b	a	8.34 (±1.53)	a	a
	8	8.77 (8.13, 9.03)	a	a	8.56 (7.99, 9.71)	a	a	8.50 (±2.06)<br>7.94 (7.09, 9.50)	a,b	a	8.59 (±1.56)<br>8.25 (7.65, 9.81)	a	a
	12	9.15 (±0.99)<br>9.14 (8.77, 9.60)	a	a	8.98 (±1.38)<br>9.34 (8.22, 9.75)	a	a	9.18 (±2.48)	b	a	8.83 (±1.41)	a	a
Cfb (%)		−8.41 (−18.86, 5.69)		a	−4.05 (−17.48, 6.99)		a	15.60 (2.07, 48.10)		b	3.16 (−7.52, 13.55)		a,b
n-6/*n*-3	0	3.24 (2.80, 3.54)	a	a	3.31 (2.93, 3.77)	a	a	4.51 (±1.25)<br>4.35 (3.56, 5.41)	a	b	3.58 (±0.68)<br>3.49 (3.05, 3.89)	a	a
	4	3.40 (±0.49)<br>3.41 (3.10, 3.70)	a,b	a	3.41 (±0.69)<br>3.40 (2.85, 3.71)	a	a	3.54 (±0.70)	b	a	3.56 (±0.72)	a	a
	8	3.50 (±0.36)<br>3.54 (3.35, 3.69)	b	a	3.47 (±0.69)<br>3.37 (2.99, 3.70)	a	a	3.75 (±0.73)	b	a	3.46 (±0.79)	a,b	a
	12	3.26 (3.11, 3.42)	a	a	3.11 (2.94, 3.70)	a	a	3.47 (±0.75)<br>3.56 (2.93, 4.00)	b	a	3.25 (±0.73)<br>3.09 (2.82, 3.60)	b	a
Cfb (%)		4.55 (±20.62)		a	4.10 (±18.09)		a	−20.64 (±14.51)		b	−8.67 (±13.62)		c

Table S3. Continued.

Erythrocyte fatty acids (% FAME)		Week	High linoleic acid (n = 27) Characteristics *	Δ	◊	Low linoleic acid (n = 27) Characteristics *	Δ	◊	High milk (n = 22) Characteristics *	Δ	◊	Control (n = 27) Characteristics *	Δ	◊
TFA		0	0.36 (0.31, 0.42)	a	a	0.33 (0.27, 0.42)	a	a	0.50 (0.44, 0.66)	a,c	b	0.48 (0.43, 0.65)	a	b
		4	0.29 (0.25, 0.33)	b	a	0.26 (0.22, 0.36)	a	a	0.53 (0.43, 0.58)	a	b	0.42 (0.39, 0.50)	a	b
		8	0.27 (0.24, 0.29)	b	a	0.27 (0.24, 0.32)	a	a	0.61 (0.49, 0.68)	b,c	b	0.49 (0.41, 0.69)	a	b
		12	0.28 (0.26, 0.53)	b	a	0.30 (0.23, 0.83)	a	a	0.68 (0.62, 0.78)	b	b	0.44 (0.38, 0.70)	a	a,b
	Cfb (%)		−14.65 (−27.85, 2.93)		a	−3.37 (−17.04, 133.81)		a,b	29.58 (12.71, 51.89)		b	−0.23 (−26.45, 34.81)		a
EPA/ALA (n † (Control) = 26)		0	6.04 (4.59, 8.45)	a	a	5.36 (4.58, 7.03)	a	a	4.60 (4.23, 6.28)	a	a	5.59 (4.20, 6.90)	a	a
		4	2.32 (1.65, 3.17)	b	a	2.17 (1.58, 2.98)	b	a	1.98 (1.65, 2.59)	b	a	2.01 (1.77, 2.53)	b	a
		8	2.15 (1.54, 2.65)	b	a	1.84 (1.45, 2.30)	b	a	1.84 (1.35, 2.38)	b	a	1.97 (1.58, 2.98)	b	a
		12	2.05 (1.51, 2.49)	b	a	1.93 (1.48, 2.81)	b	a	2.05 (1.47, 2.90)	b	a	1.99 (1.67, 2.65)	b	a
	Cfb (%)		−68.97 (−77.97, −53.31)		a	−66.94 (−71.34, −42.33)		a	−61.15 (−71.47, −50.76)		a	−62.69 (−71.87, −54.95)		a
DPA/ALA (n † (Control) = 26)		0	18.92 (16.00, 23.70)	a	a	16.50 (12.28, 21.07)	a	a,b	16.07 (13.98, 19.27)	a	a,b	14.79 (10.55, 17.35)	a	b
		4	4.63 (3.62, 6.35)	b	a	4.65 (3.51, 6.64)	b	a	4.54 (3.70, 6.75)	b	a	4.12 (3.35, 5.46)	b	a
		8	3.85 (3.35, 5.17)	b,c	a	3.33 (2.53, 5.10)	c	a	4.11 (3.47, 5.68)	b	a	3.65 (3.15, 5.32)	b	a
		12	3.50 (2.64, 4.84)	c	a	3.77 (2.69, 5.04)	b,c	a	4.45 (3.17, 6.77)	b	a	3.86 (3.22, 4.59)	b	a
	Cfb (%)		−79.35 (−87.18, −73.49)		a	−77.89 (−85.11, −52.67)		a,b	−73.33 (−79.64, −47.07)		b	−69.49 (−77.51, −61.96)		b
DHA/ALA (n † (Control) = 26)		0	37.77 (29.63, 52.46)	a	a	27.46 (20.07, 41.56)	a	a,b	23.91 (22.08, 33.54)	a	b	28.95 (19.78, 36.77)	a	b
		4	7.32 (5.78, 12.47)	b	a	7.64 (5.64, 10.35)	b	a	7.66 (5.21, 9.96)	b	a	7.26 (5.06, 8.48)	b	a
		8	6.03 (4.82, 7.55)	c	a	5.01 (3.74, 7.77)	c	a	6.19 (3.77, 9.75)	b	a	6.06 (4.78, 8.40)	b	a
		12	4.94 (3.88, 7.38)	c	a	5.37 (3.36, 7.12)	c	a	5.37 (4.08, 7.17)	b	a	5.33 (4.64, 7.14)	b	a
	Cfb (%)		−86.19 (−90.95, −81.25)		a	−85.59 (−90.36, −64.30)		a,b	−81.50 (−84.64, −61.92)		b	−77.90 (−84.20, −71.06)		b
ARA/LA		0	1.54 (1.37, 1.86)	a	a	1.50 (±0.25)<br>1.52 (1.31, 1.72)	a	a	1.29 (±0.19)<br>1.30 (1.18, 1.44)	a	b	1.35 (1.23, 1.41)	a	b
		4	1.14 (1.05, 1.40)	b	a,b	1.28 (±0.22)<br>1.30 (1.13, 1.38)	b	a	1.27 (±0.22)<br>1.27 (1.10, 1.42)	a	a	1.09 (1.04, 1.21)	b	b
		8	1.04 (0.91, 1.19)	c	a	1.10 (±0.15)<br>1.11 (0.97, 1.19)	c	a,b	1.24 (±0.22)<br>1.24 (1.07, 1.34)	a,b	b	1.09 (1.01, 1.20)	b	a,b
		12	1.07 (±0.20)<br>1.06 (0.89, 1.16)	c	a	1.10 (±0.15)	c	a	1.16 (±0.20)	b	a	1.04 (±0.17)<br>1.07 (0.93, 1.14)	c	a
	Cfb (%)		−31.11 (−39.67, −25.41)		a	−26.74 (−35.02, −18.09)		a,c	−11.87 (−16.17, −2.90)		b	−20.07 (−25.74, −15.07)		c

Table S3. Continued.

Erythrocyte fatty acids (% FAME)	Week	High linoleic acid (n = 27) Characteristics *	Δ	◊	Low linoleic acid (n = 27) Characteristics *	Δ	◊	High milk (n = 22) Characteristics *	Δ	◊	Control (n = 27) Characteristics *	Δ	◊
n-3 index	0	6.71 (±1.63)<br>6.63 (5.51, 7.53)	a	a	6.16 (5.14, 7.02)	a	a	4.84 (3.56, 5.96)	a	b	5.92 (±1.15)<br>5.85 (4.98, 6.89)	a	a,b
	4	5.74 (±1.13)	b	a	5.63 (±1.31)<br>5.42 (4.73, 6.40)	b	a	5.56 (±1.38)<br>5.25 (4.68, 6.65)	a	a	5.30 (±1.27)	b	a
	8	5.25 (±0.74)<br>5.17 (4.76, 5.65)	c	a	5.08 (4.41, 5.89)	b	a	4.63 (3.91, 5.43)	a	a	5.38 (±1.32)<br>5.11 (4.64, 6.32)	b	a
	12	5.39 (±0.81)	b,c	a	5.18 (±0.94)<br>5.25 (4.47, 5.93)	b	a	5.15 (±1.48)<br>4.98 (4.32, 6.17)	a	a	5.37 (±1.16)	b	a
Cfb (%)		−18.78 (−28.56, −11.48)		a	−21.39 (−28.81, −6.68)		a	0.69 (−9.13, 28.30)		b	−9.61 (−19.36, −2.25)		a,b

* Variables expressed as mean (±SD) and/or as median (25th, 75th percentile) depending on the statistical tests that were performed; ◊ groups without a common letter are significantly different, $p < 0.05$; Δ points in time without a common letter are significantly different, $p < 0.05$; † number of data sets used to calculate shown data if differing from n. Abbreviations: ALA, α-linolenic acid; ARA, arachidonic acid; Cfb (%), percentage change from baseline; DHA, docosahexaenoic acid; DPA, docosapentaenoic acid; EPA, eicosapentaenoic acid; ETA, eicosatetraenoic acid; FAME, fatty acid methyl ester; LA, linoleic acid; MUFA, monounsaturated fatty acids; PUFA, polyunsaturated fatty acids; SFA, saturated fatty acids; TFA, trans-fatty acids

Table S4. Comparison of percentage change from baseline without diet group subdivision but with split by sex differences, EPA baseline status and LA change throughout the study.

**Subgroups**	**Whole study collective**<br>**Change from baseline (%) ***	Δ
	C-20:5n3 (EPA)<br>(*n* † = 68, 35, 54, 49, 52, 51)	
Women	39.39 (12.85, 79.04)	
Men	69.00 (34.96, 94.35)	0.043
EPA (<0.9)Baseline	79.28 (45.72, 111.29)	
EPA (≥0.9)Baseline	28.96 (8.61, 58.01)	<0.001
LA (<1.5)Change	58.02 (26.25, 98.35)	
LA (≥1.5)Change	39.40 (16.93, 81.19)	n.s.
	n-3 index<br>(*n* † = 68, 35, 54, 49, 52, 51)	
Women	−13.33 (−27.62, 0.32)	
Men	−10.41 (−18.16, −2.25)	n.s.
EPA (<0.9)Baseline	−4.90 (−19.79, 8.21)	
EPA (≥0.9)Baseline	−17.59 (−26.72, −10.40)	<0.001
LA (<1.5)Change	−6.53 (−14.16, 7.86)	
LA (≥1.5)Change	−21.50 (−29.36, −10.41)	0.001

* Variables expressed as mean (±SD) and/or as median (25th, 75th percentile) depending on the statistical test that was performed; † Number of data sets used to calculate shown data per subgroup; Δ Comparison of subgroups; Baseline Subgroup built by using baseline EPA status (<0.9 % FAME vs. ≥0.9 % FAME); Change Subgroup built by using total LA change (% FAME) that was observed throughout the study (<1.5 % FAME vs. ≥1.5 % FAME). Abbreviations: EPA, eicosapentaenoic acid; LA, linoleic acid
